# Long‐term drought effects on the thermal sensitivity of Amazon forest trees

**DOI:** 10.1111/pce.14465

**Published:** 2022-10-20

**Authors:** Emma M. Docherty, Emanuel Gloor, Daniela Sponchiado, Martin Gilpin, Carlos A. D. Pinto, Haroldo M. Junior, Ingrid Coughlin, Leandro Ferreira, João A. S. Junior, Antonio C. L. da Costa, Patrick Meir, David Galbraith

**Affiliations:** ^1^ Department of Earth and Environment, School of Geography University of Leeds Leeds UK; ^2^ Departamento de Ciências Biológicas, Laboratório de Ecologia Vegetal Universidade do Estado de Mato Grosso Nova Xavantina Mato Grosso Brasil; ^3^ Instituto de Geosciências Universidade Federaldo Pará Belém Pará Brasil; ^4^ Departamento de Biologia, FFCLRP Universidade de São Paulo Ribeirao Preto São Paulo Brasil; ^5^ College of Science, Research School of Biology Australian National University Canberra Australian Capital Territor Australia; ^6^ Museu Paraense Emílio Goeldi Belém Pará Brasil; ^7^ College of Science and Engineering, School of GeoSciences University of Edinburgh Edinburgh UK

**Keywords:** Amazon rainforest, chlorophyll *a* fluorescence, drought and heat stress interactions, photosynthesis, respiration, thermal traits, thermotolerance, throughfall exclusion, tropical evergreen trees

## Abstract

The continued functioning of tropical forests under climate change depends on their resilience to drought and heat. However, there is little understanding of how tropical forests will respond to combinations of these stresses, and no field studies to date have explicitly evaluated whether sustained drought alters sensitivity to temperature. We measured the temperature response of net photosynthesis, foliar respiration and the maximum quantum efficiency of photosystem II (*F*_v_/*F*_m_) of eight hyper‐dominant Amazonian tree species at the world's longest‐running tropical forest drought experiment, to investigate the effect of drought on forest thermal sensitivity. Despite a 0.6°C–2°C increase in canopy air temperatures following long‐term drought, no change in overall thermal sensitivity of net photosynthesis or respiration was observed. However, photosystem II tolerance to extreme‐heat damage (*T*_50_) was reduced from 50.0 ± 0.3°C to 48.5 ± 0.3°C under drought. Our results suggest that long‐term reductions in precipitation, as projected across much of Amazonia by climate models, are unlikely to greatly alter the response of tropical forests to rising mean temperatures but may increase the risk of leaf thermal damage during heatwaves.

## 1 INTRODUCTION

Amazon forests have experienced an increasing trend in air temperature of up to 0.5°C per decade over the past 35 years (Fauset et al., 2018; I. Harris et al., 2014). These temperature increases are predicted to continue, accompanied by a potential four‐fold uptick in the frequency of heatwaves, with the tropics departing from historical temperature limits sooner than other biomes (Coumou & Robinson, 2013; Meehl & Tebaldi, 2004; Mora et al., 2013). Warmer conditions are likely to occur increasingly in combination with longer and more severe droughts across much of Amazônia (Allen et al., 2015; Marengo et al., 2018; Toomey et al., 2011). The response of forests to these changes in climate will depend on their capacity to acclimate to changing baseline environmental conditions and resilience to extreme stress (Corlett, 2016; Galbraith et al., 2010; Geange et al., 2021; Smith & Dukes, 2013; Sterck et al., 2016). Crucially, forest tree species may respond differently to heat and drought stress and this will likely influence changes in species composition, vegetation density and forest ability to sequester carbon (da Costa et al., 2010; Esquivel‐Muelbert et al., 2019). Currently, there are limited data available to aid understanding of tropical forest sensitivity to increasing temperatures. The few studies to date suggest that gas exchange processes and photosynthetic thermotolerance in tropical species are capable of some degree of thermal acclimation, however, considerable variation in both baseline thermal sensitivities and acclimation potential exists amongst species (Atkin & Tjoelker, 2003; Carter et al., 2020, 2021; Drake et al., 2016, 2018; Slot & Winter, 2017b, 2017c; Slot et al., 2014; Tiwari et al., 2021). Very few studies have examined how drought might affect tropical forest thermal sensitivity (Geange et al., 2021; Sastry et al., 2018). This represents an important knowledge gap, as research on crop species suggests the effect of simultaneous heat and drought stress on plant productivity, tissue damage and mortality are not necessarily predictable based on sensitivity to drought or heat stress alone (Rizhsky et al., 2004; Zandalinas et al., 2018).

**Figure 1:**
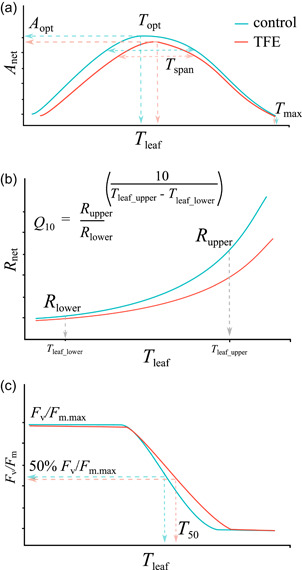
Hypothetical examples of the temperature response of net photosynthesis (*A*_net_) (a), foliar respiration in the dark (*R*_net_) (b), and maximum quantum efficiency of photosystem II (*F*_v_/*F*_m_) (c), in the control (blue) and the TFE (red), if long‐term drought were to induce thermal acclimation in these processes. TFE, through‐fall exclusion. [Color figure can be viewed at wileyonlinelibrary.com]

The thermal sensitivity of a plant is often described using properties (herein referred to as thermal traits) that confer information about the stability of gas exchange processes and/or the integrity of photosynthetic machinery under moderate to extreme heat. For example, *T*_opt_ describes the optimum temperature for photosynthesis, whilst *T*_span_ describes the breadth of temperature over which photosynthesis rates are sustained >80% of optimum rates, and *T*_max_, the high end of the temperature range within which a leaf is able to assimilate CO_2_ (Slot & Winter, 2017b) (Figure 1a, Table 1). The temperature sensitivity of respiration is usually inferred through comparing basal rates at a standard temperature (25°C, *R*_25_), and the steepness of the instantaneous increase in respiration rates over a 10°C rise in temperature (*Q*_10_) (Atkin & Tjoelker, 2003) (Figure 1b, Table 1). The above traits can be used to evaluate the capacity of leaves to maintain a positive carbon balance under rising temperatures, some of which form integral components of global vegetation models (Booth et al., 2012; Cox et al., 2000; Galbraith et al., 2010). Alternatively, the temperature at which the maximum quantum efficiency of photosystem II (*F*_v_/*F*_m_) is reduced to 50% of its value under non‐stressed conditions (*T*_50_) (Figure 1c, Table 1) is a measure of a plant's ability to protect the integrity of photosystem II (PSII) at high temperatures (Figueroa et al., 2003; Lípová et al., 2010; Zhang et al., 2012). *T*_50_ provides thermotolerance estimates comparable to those gained from classical leaf necrosis tests with the same temperature exposure time (Krause et al., 2010). Thus, it is a relevant metric for considering the potential for short periods of extreme temperature stress to impact carbon assimilation.

**Table 1:** Abbreviations and descriptions

Variable	Units	Description
*A*_net_	µmol m^−2^ s^−1^	Net photosynthesis rate
*A*_opt_	µmol m^−2^ s^−1^	Net photosynthesis rate at *T*_opt_ of *A*_net_
ETR	µmol m^−2^ s^−1^	Linear electron transport rate
*F*_v_/*F*_m_	Unitless	Maximum quantum efficiency of photosystem II chlorophyll fluorescence
*g*_s_	mol m^−2^ s^−1^	Stomatal conductance to water vapour
*g*_sdiff_	mol m^−2^ s^−1^	Difference between *g*_s*T*opt_ and *g*_sTL46_
*g*_sTL46_	mol m^−2^ s^−1^	Stomatal conductance at *T*_leaf_ = 46°C (46°C = mean *T*_max_ for this study)
*g*_s*T*opt_	mol m^−2^ s^−1^	Stomatal conductance at *T*_opt_ of *A*_net_
PSII	‐	Photosystem II
*Q*_10_	Unitless	Factor of *R*_net_ increase for every 10°C increase in leaf temperature from a reference leaf temperature
*R*_net_	µmol m^−2^ s^−1^	Net respiration rate in the dark
*R*_25_	µmol m^−2^ s^−1^	Respiration rate at *T*_leaf_ 25°C
*R*_45_	µmol m^−2^ s^−1^	Respiration rate at *T*_leaf_ 45°C
*T*_leaf_	°C	Leaf temperature
*T*_max_	°C	High‐end temperature at which *A*_net_ reaches zero
*T*_opt_	°C	Optimum temperature for *A*_net_
*T*_optETR_	µmol m^−2^ s^−1^	Optimum temperature for electron transport rate
*T*_sm_	°C	Thermal safety margin; the difference between maximum *T*_leaf_ and *T*_50_
*T*_span_	°C	Temperature range over which *A*_net_ is maintained >80% of *A*_opt_
*T*_50_	°C	Temperature at which *F*_v_/*F*_m_ is reduced by 50% relative to its value under non‐stressed conditions

The way in which combinations of drought and heat affect plant physiological processes is poorly understood, with most knowledge gained from crop species (Geange et al., 2021). However, there is a consensus that drought exacerbates temperature stress by restricting evaporative cooling and increasing leaf temperatures (Suzuki et al., 2014). Short‐term coupled heat and drought events, such as those experienced during hot, dry El Niño years, have been linked to reduced tropical forest productivity, contributing to a weaker forest carbon sink (Cavaleri et al., 2017). However, long‐term (multi‐year to decadal) reductions in precipitation might be expected to interact differently with thermal stress, as plants have more time to employ structural and/or metabolic rate adjustments to sustained water limitation. For instance, net photosynthesis, respiration and leaf thermotolerance might thermally acclimate (i.e., adjust to maintain a positive carbon balance and thermal safety margin) to a sustained drought‐induced rise in leaf temperatures (Figure 1) (Atkin & Tjoelker, 2003; Berry & Bjorkman, 1980; Way & Yamori, 2014). Thermal acclimation has been observed in leaf respiration (downregulation of *R*_25_ and *Q*_10_) and thermotolerance (upregulation of *F*_v_/*F*_m_ at 47.5°C) metrics after relatively short (weeks to months) experimental droughts on tropical saplings (Gauthier et al., 2014; Sastry et al., 2018). Although not previously evaluated in relation to drought, and inconsistent across species, photosynthetic (upregulation of *T*_opt_, *T*_span_ and *T*_max_) and respiratory acclimation has been observed in warming studies on tropical saplings and in some understory shrubs (Carter et al., 2020; Slot & Winter, 2017c; Slot et al., 2014; Mujawamariya et al., 2021). Similarly, an in situ +3°C leaf warming study on two mature tropical tree species observed some photosynthetic and respiratory acclimation, though inconsistent between species and leaf canopy position (Carter et al., 2021). If thermal acclimation were widespread and sustained over long timescales, this might buffer the negative effects of heat and drought combinations on forest productivity. It has also been proposed that a partial overlap in protective mechanisms, such as altering chloroplast membrane compositions (Ladjal et al., 2000), or upregulation of antioxidant scavenging (Gill & Tuteja, 2010), could facilitate cross‐protection between drought and warming and that exposure to drought could effectively prime physiological processes for heat exposure (Havaux et al., 1988). However, metabolic profiling studies on crop species, have observed little overlap in cellular responses to drought and heat (Rizhsky et al., 2004; Zandalinas et al., 2018), and therefore prolonged drought might not alter thermal sensitivity. On the other hand, sustained drought may restrict plant capacity to deal with stress‐by‐products (Gill & Tuteja, 2010), or cellular maintenance and repair. This would weaken plant ability to cope with additional heat stress, due to limited scope for additional upregulation, or because substrate reserves that would otherwise build‐up under non‐stress conditions become depleted (Shaar‐Moshe et al., 2019). Furthermore, sustained tissue damage under long‐term drought—for example, damage of the plant water transport system, may also constrain physiological processes so that they are less able to adjust to compounding adverse conditions, such as leaf transpirational cooling in response to heat (Rehschuh et al., 2020; Skelton et al., 2017).

In tropical forests where biodiversity is high, both drought and thermal sensitivity (and their acclimation potentials) have been shown to vary markedly amongst co‐occurring species (Bittencourt et al., 2020; da Costa et al., 2010; Perez & Feeley, 2020; Rowland, Lobo‐do‐Vale, et al., 2015; Sastry & Barua, 2017; Slot & Kitajima, 2015; Slot & Winter, 2017b). Owing to the interaction between drought and heat, the effect of sustained drought on species thermal sensitivities will likely also vary. There is some evidence that physiological responses to drought and temperature stress might be coordinated. For instance, short‐term drought tolerance (measured via leaf wilting), has been positively related to heat tolerance in saplings of 12 seasonally dry tropical forest tree species (Sastry et al., 2018). Understanding the extent to which this is true for adult tropical forest trees would help identify sensitive taxa and potential alterations in community composition, therefore advancing predictive insights of future ecosystem functioning.

In this study, we use a longstanding (17‐year) rainfall exclusion experiment to evaluate the effect of long‐term reduction in soil water availability on thermal sensitivity traits in an old‐growth tropical forest in eastern Amazonia. Additionally, we compare thermal traits across tree species that have previously been classified as drought‐tolerant or intolerant, based on their mortality response to the same experimental drought (da Costa et al., 2010; Rowland, Lobo‐do‐Vale, et al., 2015). We test whether the direction and magnitude of thermal trait adjustment in response to sustained drought treatment is influenced by drought tolerance, and if there is coordination, independent of treatment, between drought and thermal sensitivity. Using thermal trait measurements linked to photosynthesis, leaf respiration and thermotolerance of PSII for 48 individuals, spanning eight species across control and drought plots, we address the following questions:

1) Does long‐term drought alter tropical forest thermal sensitivity?
2) Are drought‐intolerant species more sensitive to heat stress compared to drought‐tolerant species?
3) Do drought‐intolerant and drought‐tolerant species adjust their thermal traits differently to long‐term drought?

## 2 METHODS

### 2.1 Study site

The study was undertaken at a long‐term through‐fall exclusion (TFE) experiment, located within a tropical evergreen forest in the Caxiuanã National Forest Reserve, eastern Amazonia, Brazil (1˚43'S, 51˚27'W). The TFE experiment was established in 2002, and consists of two 1‐ha plots, a ‘control’ and ‘TFE’ (treatment), within an old‐growth *terra firme* forest. A network of plastic panels and guttering at a height of 1–2 m excludes 50% of incoming rainfall from reaching the soil in the TFE plot, whilst the control plot receives no rainfall manipulation. The perimeter of both plots is trenched to reduced lateral water inflow from outside of the plot area. Mean annual air temperature at the site is 25.9°C, with minimum and maximums of 20.7°C and 33.8°C respectively. Precipitation varies between 2000 and 2500 mm year^−1^, with a pronounced dry season from June to November.

**Figure 2:**
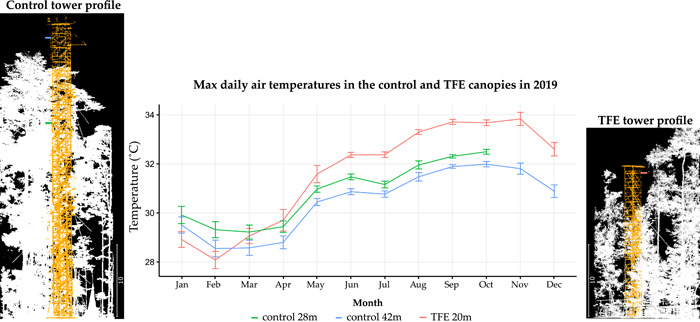
Maximum daily air temperatures in the control (green and blue) and the TFE (red) canopies at Caxiuanã, during 2019. Lines show the mean maximum daily air temperatures at 28 m (green) and 42 m (blue) height in the control, and at 20 m (red) height in the TFE, for each month. Error bars denote one standard error. Tower profiles show the position of air temperature sensors in relation to the immediately surrounding canopy heights. Plot level mean/median tree heights are 23.3/22.3 m and 21.0/20.1 m for the control and TFE respectively. From January to April maximum canopy air temperatures in the TFE did not differ from the control, likely reflecting increased TFE transpiration rates during the wet season (da Costa et al., 2018). However, from May through to December, maximum daily canopy air temperatures were consistently higher in the TFE compared to the control by 0.6°C–2°C. TFE, through‐fall exclusion. [Color figure can be viewed at wileyonlinelibrary.com]

Tree mortality rates in the TFE increased markedly 3 years after experiment installation (da Costa et al., 2010; Meir et al., 2015). Differential mortality rates across tree genera were observed after 7 years TFE exposure, leading to categorization of drought‐intolerant and tolerant genera, with intolerant genera being those experiencing >1.5 times higher mortality rates in the TFE relative to the control (da Costa et al., 2010; Rowland, Lobo‐do‐Vale, et al., 2015). Following 14 years of sustained drought, aboveground biomass in the TFE had reduced by 40%, increasing light interception in the lower canopy (Rowland, da Costa, et al., 2015). Despite the consequent reduction in inter‐tree competition for soil water, TFE trees have been estimated from sap flux data to transpire close to 100% of the rain through‐fall available to them, compared to 75% of the rainfall in the control (da Costa et al., 2018); leaf water potentials and branch hydraulic conductivity remain lower in the TFE relative to control; and soil water content remains significantly lower in the TFE relative to the control, indicating that soil water stress has not been alleviated for surviving trees (Bittencourt et al., 2020). Analysis of locally‐collected air temperature data in both plots showed that daily maximum canopy air temperatures in 2019 were 0.6°C–2°C higher in the TFE relative to the control, from May through to December (Figure 2). For further information on experimental design and results see Meir et al. (2018), Rowland, da Costa, et al. (2021) and references within.

### 2.2 Plant material

Measurements were performed at the beginning of the dry season, between 08 June and 08 July 2019, on 48 mature trees, comprising eight of the most dominant (in terms of basal area) species within the control plot, that also had at least three individuals remaining in the TFE. Together these species represent 20% of the control plot basal area. Four of these species were previously categorized as drought‐tolerant and four as drought‐intolerant (see definition in Section 2.1) by da Costa et al. (2010) and Rowland, Lobo‐do‐Vale, et al. (2015) (Table 2). Additionally, all study species belong to genera ranked within the top 103 most abundant Amazonian genera, with *Eschweilera*, *Pouteria*, *Licania* and *Swartzia* ranking within the top 20 (Ter Steege et al., 2013). One temperature response curve for photosynthesis, respiration and thermotolerance was measured on three mature individuals per species, in each plot, ensuring even DBH (Diameter at Breast Height) representation between both treatment plots and drought‐tolerance status. Using telescopic shears, trained climbers excised fully sun exposed, upper canopy branches (~1 m length) before 07:00 h for respiration and thermotolerance assays (except for six branches that were collected before 09:30 h), and between 08:00 and 12:00 h for photosynthesis measurements. Maximum vessel lengths for the species studied at this site are on average 32.7 ± 15.2 cm (55.5 cm maximum) (Bittencourt et al., 2020; Rowland, da Costa, et al., 2015). Accordingly, ~1 m branch lengths were considered sufficient to avoid open vessel artifacts in measurements. Separate branches were used for photosynthesis, respiration and thermotolerance curves. Upon reaching the ground, harvested branch stems were immediately cut underwater to maintain hydraulic connectivity in the xylem, wrapped in a water‐soaked cloth and transferred a short distance to the research station, where they were again re‐cut under water. For all measurements, we selected fully expanded healthy leaves, avoiding herbivory and fungus. Leaves selected for respiration measurements were covered in aluminium foil and branches for both respiration and thermotolerance were kept in buckets of water in the shade until measurements could be performed, typically within 2 h for thermotolerance and 4–13 h for respiration. Photosynthesis branches were kept in buckets of water in full sun, and measurements started within 20 min of arrival at the research station. Neither the variation in collection time or time‐delay between collection and respiration assays influenced measured gas exchange traits (Supporting Information: Figure S1). Individual temperature response curves for photosynthesis, respiration and thermotolerance took on average 3 h to complete.

**Table 2:** Summary of the species for which thermal traits were measured in both the control and through‐fall exclusion plots at Caxiuanã and their drought tolerance as defined by da Costa et al. (2010) and Rowland, Lobo‐do‐Vale, et al. (2015)

Species^a^	Drought tolerance
*Licania octandra* b	Tolerant
*Minquartia guianensis*	Tolerant
*Swartzia racemosa*	Tolerant
*Vouacapoua americana*	Tolerant
*Eschweilera coriacea*	Intolerant
*Manilkara bidentata* c	Intolerant
*Pouteria decorticans*	Intolerant
*Pouteria guianensis*	Intolerant

^a^ Three individuals per species, per plot.

^b^ Species was not included in any analysis for *T*_opt_, *A*_opt_, *T*_span_, *g*_s*T*opt_ and *g*_sdiff_ as the shape of *A‐T* curves in individuals from the control plot meant that it was not possible to extract these parameters (Supporting Information: Figure S2, Supporting Information: Methods S1).

^c^ Only thermotolerance measurements were collected for this species.

### 2.3 Temperature response of net photosynthesis

Photosynthesis temperature (*A‐T*) response curves were constructed between 09:00 and 14:00 h using three infra‐red greenhouse gas analysers (two LI‐6400XT and one LI‐6800), with either LED (6400‐02B) or fluorometer (6400‐40) chamber heads (LI‐COR). LI‐COR machine use was distributed evenly across sampling (i.e., all species were measured with both LI‐6400XT and LI‐6800 machines). This enabled confirmation that the LI‐COR model used did not result in inherent biases in extracted *A‐T* traits (Supporting Information: Table S1, Supporting Information: Methods S3). Selected leaves were clipped into leaf chambers, ensuring good leaf‐to‐thermocouple contact (Supporting Information: Methods S3), and allowed to stabilize at reference CO_2_, photosynthetic photon flux density (PPFD), relative humidity (RH) and ambient air temperature for at least 15 min before *A‐T* response curves were initiated. Net photosynthesis rates were measured at leaf chamber air temperatures of 23°C, 26°C, 29°C, 32°C, 35°C, 38°C, 41°C, 45°C and 50°C giving a range in leaf temperatures of 23°C–50°C (Supporting Information: Figure S2). For each temperature point, leaf temperature (*T*_leaf_), RH, stomatal conductance to water vapour (*g*_s_), leaf‐to‐air vapour pressure deficit (VPD) and photosynthetic rate (*A*_net_) were allowed to stabilize and then maintained at steady‐state for at least 8 min before recording measurements. For the two LI‐6400XT models, a water bath and accompanying temperature expansion kit were used to reach the highest temperatures (>41°C). Linear electron transport rates (ETR) derived from chlorophyll‐*a* fluorescence, were logged simultaneously with *A*_net_ and other relevant parameters in the two LI‐CORs with fluorometer chamber heads. For all measurements, reference CO_2_ was maintained at 400 ppm, representing ambient CO_2_ concentrations and PPFD at 1100 µmol m^−2^ s^−1^, representing standard light‐saturating levels. Whilst the photosynthesis of all species may not be fully saturated at this light intensity, increases in *A*_net_ above 1100 µmol m^−2^ s^−1^ are minimal for most species, and this level of light is unlikely to result in photoinhibition at low and high temperatures (Slot & Winter, 2017b). We attempted to control leaf chamber RH at 50% throughout measurements. However, at temperatures above ~40°C, this was difficult to maintain and despite the use of humidifiers RH typically dropped to between 30% and 40%. The combination of increasing chamber temperature and constant or declining RH resulted in substantial increases in VPD during the temperature range of our measurements. VPD increased on average from ~2 kPa at *T*_leaf_~25°C to ~8 kPa at *T*_leaf_~50°C. Thus, our temperature responses incorporate both the direct effects of increasing *T*_leaf_ on *A*_net_ and the indirect effects of increasing VPD on *g*_s_. Leaf temperatures and VPD are highly coupled under natural conditions and our measurements account for this coupling. Relationships between *T*_leaf_, VPD, *g*_s_ and *A*_net_ are shown in Supporting Information: Figure S3. Natural variation in the shape of *A‐T* response curves meant that no single model equation provided a good fit across all individual curves (Supporting Information: Figures S2 and S4). Accordingly, to facilitate extraction of the most precise thermal trait values, each *A‐T* curve was fit using four different equations. First, we fit the data using a standard quadratic equation as:

(1) $$A_{\text{net}}=a{T_{\text{leaf}}}^{2}+bT_{\text{leaf}}+c,$$

where *A*_net_ is net photosynthesis (μmol m^–2^ s^–1^) at *T*_leaf_ (°C), and *a*, *b* and *c* are coefficients that describe the *A‐T* response. Second, we used the June et al. (2004) equation.

(2) $$A_{\text{net}}=A_{\text{opt}}\times e^{-{\left(\frac{T_{\text{leaf}}-T_{\text{opt}}}{Ω}\right)}^{2}},$$

where *A*_opt_ is the value of *A*_net_ at the optimum temperature for photosynthesis (*T*_opt_), and Ω is the temperature difference between *T*_opt_ and the temperature at which *A*_net_ drops to e^–1^ (37%) of *A*_opt_. Equation 2 assumes that the slope of the *A‐T* response asymptotes as *A*_net_ approaches zero, such that *A*_net_ never passes through zero, which is not the case in nature. To account for this, we also used an adjusted version of the June et al. (2004) equation.

(3) $$A_{\text{net}}=A_{\text{opt}}\times e^{-{\left(\frac{T_{\text{leaf}}-T_{\text{opt}}}{Ω}\right)}^{2}}-c,$$

where *c* is a constant that allows *A*_net_ to pass through zero. Equations (1), (2), (3) all assume a symmetrical *A‐T* response around *T*_opt_, which was not the case for all species in this study. Accordingly, for those species with an asymmetrical *A‐T* response, the data were also fitted using the model of Cunningham and Read (2002).

(4) $$A_{\text{net}}=b\times \left(T_{\text{leaf}}-T_{\min}\right)\times (1-e^{c\left(T_{\text{leaf}}-T_{\max}\right)}),$$

where *T*_min_ and *T*_max_ are the low and high‐temperature CO_2_ compensation points respectively and *b* and *c* are fitting coefficients. The best‐fitting equation was determined for each *A‐T* curve using Akaike's Information Criterion (AIC). The equation with the lowest AIC value was then used to extract *T*_opt_, *A*_opt_, *T*_span_ (comparing Equations (1), (2), (3)) and *T*_max_ (comparing Equations 1, 3 and 4) for each *A‐T* curve. *T*_span_ was calculated as the temperature range over which *A*_net_ rates were >80% of *A*_opt_ (Figure 1a). Most *A‐T* curves had one equation that clearly fit best, however, for *A‐T* curves where multiple equations fit equally well (i.e., within two AIC units of the most parsimonious equation), we compared extracted parameters to confirm they provided similar values (Supporting Information: Figure S5). Furthermore, all best‐fitting equations were visually inspected to ensure that extracted parameter values were realistic.

The temperature response of *g*_s_ (*g*_s_‐*T*), derived from water vapour flux in the LI‐COR, measured in conjunction with *A*_net_, was also fitted using Equations (1), (2), (3), (4), replacing *A*_net_ with *g*_s_ in all equations. The *g*_s_‐*T* equation with the lowest AIC value was used to extract *g*_s_ rates at the *T*_opt_ of each corresponding *A‐T* curve (*g*_s*T*opt_), representing *g*_s_ rates at optimum temperatures for photosynthesis, and *T*_leaf_ = 46°C (*g*_sTL46_), representing *g*_s_ rates at high leaf temperatures (i.e., temperatures approaching *T*_max_ for most species). The mean *T*_max_ of all *A‐T* response curves (*T*_leaf_ = 46°C) was used as a standard ‘adversely high’ *T*_leaf_, rather than *T*_max_ values from individual curves, to avoid excluding a few *A‐T* response curves for which *T*_max_ was not able to be extrapolated (Supporting Information: Figures S2 and S6, Supporting Information: Methods S1). Negative *g*_s_ rates were considered biologically unrealistic, thus five *g*_sTL46_ values (representing 12% of all *g*_sTL46_ values) that were extracted from fitted *g*_s_‐*T* curves that passed below 0 before *T*_leaf_ = 46°C were replaced by 0. Notably, *g*_s_‐*T* curves did not always follow a typical bell‐shape, with some species departing from optimal stomatal behaviour (Medlyn et al., 2011), by increasing *g*_s_ as *T*_leaf_ rose, likely to facilitate leaf cooling (Supporting Information: Figures S3 and S6, Supporting Information: Methods S2). Therefore, to distinguish species across a spectrum, from those that showed a strong downregulation in *g*_s_ at high relative to optimum temperatures, to those that upregulated *g*_s_ despite adversely rising temperatures, we also calculated *g*_sdiff_, as the difference between *g*_sTL46_ and *g*_s*T*opt_. Accordingly, a higher *g*_sdiff_ denotes a greater downregulation in *g*_s_ at high temperature, relative to *T*_opt_, whilst a negative *g*_sdiff_ indicates an upregulation in *g*_s_ at high relative to optimum temperatures.

As with *A*_net_ and *g*_s_, ETR temperature (*ETR‐T*) response curves were fit using Equations 1(1), (2), (3) (Supporting Information: Figure S7), and the optimum temperature for ETR (*T*_optETR_), representing the temperature above which ETR becomes limiting for photosynthesis, was extracted from the *ETR‐T* equation with the lowest AIC value. All fitting was performed using either using the linear ‘lm’ or non‐linear least‐squares ‘nls’ functions in the ‘stats’ package in R version 4.0.0 (R Core Team, 2020).

### 2.4 Temperature response of dark respiration

Dark respiration temperature (*R‐T*) response curves were measured between 13:00 and 19:00 h using the LI‐6800 with a 2 cm^2^ leaf aperture (LI‐COR). Dark‐adapted leaves (see ‘Plant material’ section for details) were clipped into leaf chambers with the chamber light source and fluorometer measurement lights switched off, and allowed to stabilize at reference CO_2_, RH and ambient air temperature for at least 15 min before *R‐T* curves were initiated. *R‐T* curves were constructed using the same temperature increments, reference CO_2_ concentrations and RH controls as *A‐T* curves (Supporting Information: Figure S8). Respiration rates were <0.1 µmol m^–2^ s^–1^ in a few species at the lowest temperatures. Since the precision of the LI‐6800 IRGA at 400 µmol mol^–1^ is ≤0.1 µmol mol^–1^, we considered any respiration values <0.1µmol m^–2^ s^–1^ could be a result of IRGA signal noise and thus they were removed before analysis. Respiration rates at *T*_leaf_~25°C (*R*_~25_), ~30°C (*R*_~30_) and ~45°C (*R*_~45_) were extracted by pooling the three data points closest to the respective leaf temperatures and calculating the mean *T*_leaf_ and *R*_net_. The removal of respiration rates <0.1 µmol m^–2^ s^–1^ had no effect on parameter extraction other than preventing extraction of *R*_~25_ for three out of 42 individuals due to the low rates mentioned above. *Q*_10_ values were then calculated from *R*_~30_, *R*_~45_ and their respective *T*_leaf_ values as:

(5) $$Q_{10}={\left(\frac{R_{\sim 45}}{R_{\sim 30}}\right)}^{\left(\frac{10}{T_{\mathrm{leaf}\sim 45}-T_{\mathrm{leaf}\sim 30}}\right)},$$

where *R*_~30_ is respiration rate at *T*_leaf~30_ and *R*_~45_ is respiration rate at *T*_leaf~45_. *R*_25_ and *R*_45_ were then extrapolated to exactly 25°C and 45°C respectively using *R*_~25_ and *R*_~45_ and their corresponding *T*_leaf_ values as:

(6) $$R_{T}=R_{T_{\mathrm{leaf}}}\times {Q_{10}}^{\left(\frac{T-T_{\text{leaf}}}{10}\right)},$$

where *R*_T_ is respiration at temperature *T*, *R*_Tleaf_ is measured respiration rate at *T*_leaf_, and *Q*_10_ is the value estimated using Equation 5 for that individual *R‐T* curve. Whilst *R*_25_ was considered appropriate to represent basal *R* rates and maintain consistency with previous studies, *R*_~30_ as opposed to *R*_~25_ was used with *R*_~45_ to calculate all *Q*_10_ values to avoid excluding the individuals for which *R*_25_ was not able to be extracted.

### 2.5 Thermotolerance of PSII

Thermotolerance of PSII was determined from thermotolerance assays adapted from Krause et al. (2010). Leaf discs (diameter 1.6 cm) were cut from mature, healthy leaves underwater using a cork borer, avoiding the central vein. To capture a representative sample from each branch, 20 leaves were selected, and two discs cut from each leaf, except for occasions (<7%) where suitable leaf material was limited. Leaf discs were then wrapped in a thin layer of moist tissue paper and placed individually into sealable plastic bags, thereby avoiding touching leaves and ensuring leaf discs always remained coated with a thin film of water to prevent anaerobiosis during heating (G. C. Harris & Heber, 1993). Separate sets of five leaf discs were then submerged in a preheated circulating water bath (Grant Instruments Ltd.) for 15 min at the following temperatures: 30°C, 40°C, 45°C, 47°C, 50°C, 55°C, 60°C, with one untreated set as a control. Post‐treatment, leaf discs were placed, in their plastic bags, into opaque tubs with water at room temperature (~27°C) for at least 30 min dark adaptation, before measuring *F*_v_/*F*_m_ with a FluorPen FP100 (Photon System Instruments) (for details of FP100 fluorescence pulse specifications see Tiwari et al., 2021). For each *F*_v_/*F*_m_ temperature response assay, a three‐parameter logistic curve was fitted as:

(7) $$F_{v}/F_{m}=\frac{F_{v}/F_{m.\max}}{1+e^{b(T_{\text{leaf}}-T_{50})}},$$

where *F*_v_/*F*_m.max_ is the upper asymptote, *b* is the slope of the decrease in *F*_v_/*F*_m_ with rising temperature, *T*_leaf_ is the leaf treatment temperature, and *T*_50_ is the inflection point or temperature at which *F*_v_/*F*_m_ drops to 50% of *F*_v_/*F*_m.max_ (Supporting Information: Figure S9). Curves were fitted using ‘nlsLM’, a modified non‐linear least squares function that incorporates the Levenberg Marquardt type fitting algorithm (Moré, 1978), in the ‘minpack.lm’ package (Elzhov et al., 2016) in R.

### 2.6 Statistical analysis

Mixed effect model analysis, using the ‘lme4’ package (Bates et al., 2015) in R version 4.0.0 (R Core Team, 2020), was conducted to test for differences between control and TFE plots, and between drought‐tolerant and intolerant species, for all thermal traits separately (see Dataset S1 for the full data set used in this study). Mixed models were constructed with treatment (plot) and drought‐tolerance status (tolerant/intolerant) as fixed effects and species as random effects. To test whether thermal traits of drought‐tolerant and intolerant species were differently affected by long‐term drought, the same analysis was performed on the data separated by drought‐tolerance status with treatment (plot) as the fixed affect and species as the random effect. These models were run separately rather than including their interaction in the main model as our relatively low sample size prevented robust detection of interaction effects (Leon & Heo, 2009). Each model was tested for non‐constant error variance using the *check_heteroscedasticity* function from the *performance* package (Lüdecke et al., 2021). If error variance was deemed heteroscedastic, the response variable was log‐transformed (Supporting Information: Tables S2 and S3), and the model ran again. Log‐transformation did not resolve heteroscedasticity in model error variance for *T*_optETR_ and so an equivalent non‐parametric Type III Walt *F* test with Kenward‐Roger df was performed using the *ARTool* package (Elkin et al., 2021; Wobbrock et al., 2011) for this parameter. Due to the logistical difficulties of sampling, high species diversity and consequent relatively low replication of each thermal trait at the species level (*n* ≤ 3 per plot), we did not test for differences in thermal traits between control and TFE for individual species.

## 3 RESULTS

### 3.1 TFE effects on thermal traits

**Figure 3:**
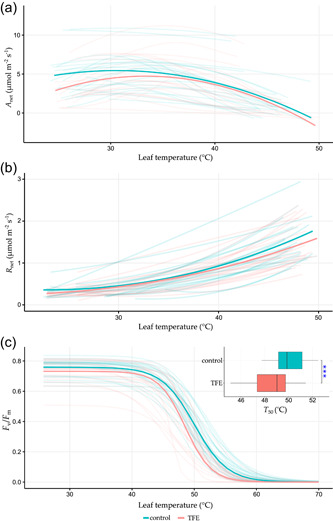
Temperature response curves of *A*_net_ (a), *R*_net_ (b) and *F*_v_/*F*_m_ (c) for control (blue lines) and TFE (red lines) plots at Caxiuanã. Bold lines show averaged temperature response curves for each plot and faded lines show individual temperature response curves. Boxplots within subplot c also demonstrate the range of *T*_50_ values within the control (blue) and the TFE (red) plots. Boxes represent 25–75 percentiles, lines within boxes are medians, and whisker lines show 10–90 percentiles. Blue stars indicate a significant difference (*P =* 1.40e^−4^), between the control and TFE plots from mixed effects analysis (see Section 2.6). TFE, through‐fall exclusion. [Color figure can be viewed at wileyonlinelibrary.com]

Overall, there were slight shifts in the shape of the average temperature response of *A*_net_, and *R*_net_ in TFE relative to the control in the direction of a thermal acclimation (i.e., *T*_opt_ displacement to the right and downward shift in *R*_net_ at high temperatures [Figure 3]). However, variation amongst temperature response curves meant that no significant TFE effect was evident for any thermal traits relating to *A*_net_, *g*_s_, ETR or *R*_net_ (Figure 3, Supporting Information: Figure S10). Conversely, thermotolerance of PSII (*T*_50_) in the TFE was significantly (*P =* 1.40e^−4^) lower (48.5 ± 0.35°C) (mean ± 1 SE) than the control (50.0 ± 0.25°C) (Figure 3).

### 3.2 Coordination of drought and thermal sensitivity

**Figure 4:**
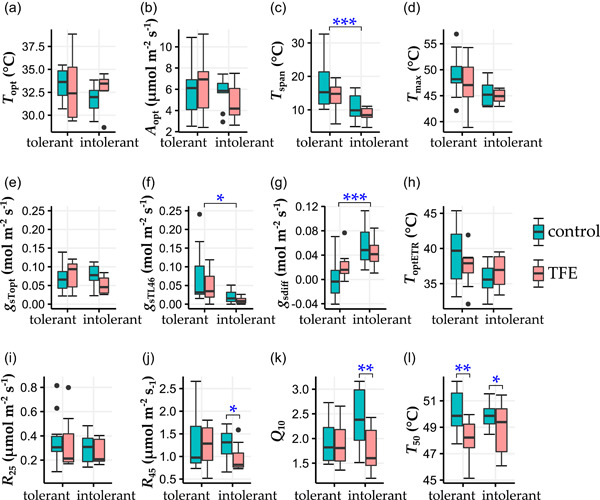
Boxplots of *T*_opt_ (a), *A*_opt_ (b), *T*_span_ (c), *T*_max_ (d), *g*_s*T*opt_ (e), *g*_sTL46_ (f), *g*_sdiff_ (g), *T*_optETR_ (h), *R*_25_ (i), *R*_45_ (j), *Q*_10_ (k) and *T*_50_ (l) for drought‐tolerant and drought‐intolerant species, in the control (blue) and the TFE (red). Boxes show 25–75 percentiles, vertical lines show 10–90 percentiles, horizontal lines within boxes are medians, and points outside the boxes represent outliers. Blue stars indicate significant differences from mixed effects analysis (see Section 2.6), either between the control and TFE plots within different drought tolerance groupings, when associated with narrow lines or between drought‐tolerant and intolerant species, irrespective of treatment, when associated with wide lines. TFE, through‐fall exclusion. [Color figure can be viewed at wileyonlinelibrary.com]

Independent of treatment, drought‐intolerant species had 38% lower *T*_span_, 73% lower *g*_sTL46_ and five‐fold higher *g*_sdiff_ compared to drought‐tolerant species (drought‐intolerant *T*_span_ = 9.6 ± 0.7°C, drought‐tolerant *T*_span_ = 15.6 ± 1.3°C, *P =* 2.46e^−4^; drought‐intolerant *g*_sTL46_ = 0.016 ± 0.003 mol m^−2^ s^−1^, drought‐tolerant *g*_sTL46_ = 0.059 ± 0.012 mol m^−2^ s^−1^, *p =* 0.01; drought‐intolerant *g*_sdiff_ = 0.049 ± 0.006 mol m^−2^ s^−1^, drought‐tolerant *g*_sdiff_ = 0.01 ± 0.006 mol m^−2^ s^−1^, *P =* 7.50e^‐4^; Figure 4). There were no significant differences between drought‐intolerant and drought‐tolerant species for any other thermal traits measured.

### 3.3 Differing TFE effects on drought‐tolerant and intolerant species thermal traits

After separating drought‐tolerant and drought‐intolerant species, there was still no TFE effect on gas exchange traits for drought‐tolerant species, but drought‐intolerant species showed significant reductions in *R*_45_ (*p =* 0.03) and Q_10_ (*p =* 0.008) in the TFE relative to the control. Drought‐intolerant species *R*_45_ was 31% lower in the TFE (0.97 ± 0.087 µmol m^−2^ s^−1^) relative to the control (1.27 ± 0.101 µmol m^−2^ s^−1^) (Figure 4e). Similarly, *Q*_10_ of drought‐intolerant species was 29% lower in the TFE (1.74 ± 0.13) relative to the control (2.24 ± 0.18) (Figure 4k). In contrast, the reductions in thermal tolerance observed overall in the TFE occurred in both drought‐tolerant (*p =* 0.002) and drought‐intolerant (*p =* 0.03) species. However, the magnitude of the drought‐associated reduction in *T*_50_ in drought‐tolerant species was double that compared to drought‐intolerant species, with reductions of 2.06 ± 0.6°C (4%) and 0.94 ± 0.6°C (2%) respectively (Figure 4l).

## 4 DISCUSSION

### 4.1 TFE effects on thermal traits overall

Based on an evaluation of leaf thermal traits for 48 mature trees covering eight hyper‐dominant Amazonian species (Ter Steege et al., 2013), we find no evidence that long‐term soil water stress (Bittencourt et al., 2020), and an accompanying drought‐induced rise in canopy air temperatures (Figure 2), alters the average thermal sensitivity of *A*_net_ or *R*_net_ across the species measured in this study. However, we do observe a moderate weakening in leaf tolerance to extreme‐heat damage, evidenced by a 1.5 ± 0.4°C reduction in mean *T*_50_. Whilst there was some variation in the magnitude of *T*_50_ response to TFE conditions amongst species, there was no indication that TFE conditions increased *T*_50_ in any species (Supporting Information: Figure S11). The lack of thermal acclimation in gas exchange traits implies that either: (a) TFE leaf temperatures were not sufficiently raised or sufficiently raised for long enough (see monthly variation in maximum daily air temperatures in Figure 2), to elicit substantial acclimation responses, (b) the species measured have limited ability to thermally acclimate to any resultant increases in *T*_leaf_, or (c) the prolonged exposure to drought stress prevented, rather than promoted thermal acclimation. We do not have detailed long‐term leaf temperature data to definitively rule out the first possibility (that leaf temperatures were not increased). However, the observed increase in canopy air temperatures for much of the year, including the month before measurements (Figure 2), combined with lower dry‐season sap flow rates in the TFE relative to the control (da Costa et al., 2018), a lack of difference in leaf mass per area across plots (Rowland, Oliveira, et al., 2021), and expectations based on leaf energy balance considerations (Fauset et al., 2018) all imply higher TFE leaf temperatures. Thus, we expect that the second (limited ability to thermally acclimate) or third (drought‐prevented thermal acclimation) possibilities are more likely to explain our results. The lack of observed acclimation in gas exchange contradicts previous studies that have shown strong thermal acclimation in tropical saplings (Mujawamariya et al., 2021; Slot & Winter, 2017c; Slot et al., 2014). However, it provides some support to studies observing limited capacity of tropical species to acclimate to warming (Carter et al., 2020, 2021). For example, after ~1 month of continuous +3°C leaf warming, Carter et al. (2021) observed no photosynthetic acclimation in upper canopy leaves and respiratory acclimation in only one of two adult tropical tree species studied.

The weakened thermotolerance in the TFE relative to the control also contrasts with findings from short‐term drought studies (Sastry et al., 2018). Temperature‐induced *F*_v_/*F*_m_ decline is associated with a breakdown of the integrity of PSII, which can occur because of a build‐up of excess heat energy and/or stress by‐products (e.g., reactive oxygen species), that interfere with thylakoid membrane stability, causing disruptions and eventual disassembly of the light‐harvesting antenna complex from the core of PSII (Figueroa et al., 2003; Lípová et al., 2010; Zhang et al., 2012). Short‐term drought stress can stimulate antioxidant production to deal with stress by‐products (Gill & Tuteja, 2010), which might explain why short‐term drought has been found to benefit thermotolerance (Sastry et al., 2018). Conversely, sustained drought, as investigated in this study, might deplete antioxidants and their substrates, therefore reducing capacities to deal with heat stress. Similarly, there is evidence that isoprene emission, a thermo‐protective trait held by several species in this study (Jardine et al., 2020; Taylor et al., 2018), can be upregulated to lessen oxidative damage to photosynthetic machinery during short‐term drought (Ryan et al., 2014; Tattini et al., 2015; Velikova et al., 2016). However, isoprene synthesis is carbon intensive (Fang et al., 1996; Sharkey & Loreto, 1993; Tattini et al., 2015), and so may be less advantageous under regular (Taylor et al., 2018), or sustained water stress when CO_2_ assimilation is already strained; potentially impeding the ability to protect photosynthetic machinery during subsequent extreme heat (Fortunati et al., 2008). Additionally, some proteins that provide protective functions during leaf desiccation (e.g., LEA), might interfere with the function of heat‐shock proteins that otherwise help maintain membrane stability under high temperatures (Rizhsky et al., 2004; Soulages et al., 2002).

### 4.2 Coordination of drought tolerance and thermal sensitivity

Independent of treatment, the *T*_span_ of drought‐tolerant species was on average, 6.0 ± 1.7°C wider than drought‐intolerant species (Figure 4c). This indicates that drought‐tolerant species are more able to maintain high rates of photosynthesis over wide temperature ranges, and therefore, any rise in mean air temperatures would result in a smaller proportional reduction in *A*_net_ for drought‐tolerant, compared to drought‐intolerant species. Similarly, *g*_s_ at high temperatures (*g*_sTL46_) and downregulation in *g*_s_ at high temperature relative to optimum leaf temperatures (*g*_sdiff_) were *c*. four times lower and five times greater in drought‐intolerant relative to drought‐tolerant species respectively (Figure 4f,g), indicating stomatal conductance in drought‐tolerant species is less sensitive to high temperatures compared to drought‐intolerant species. The fact that drought‐tolerant species had lower *g*_sdiff_ combined with a wider *T*_span_ tends to disagree with the theory presented by Michaeltz et al., (2016) that species that maintain tighter leaf temperature regulation have a narrower *T*_span_. However, whilst a lower *g*_sdiff_ will influence *T*_leaf_ by improving transpirational cooling at high temperatures (Suzuki et al., 2014), *g*_s_ only provides a small contribution, relative to other leaf traits, to the thermal time constant used to infer overall leaf thermal stability (Michaletz et al., 2016). Moreover, a reduction in *g*_s_ at high temperatures has been recognized to negatively influence *T*_span_ in other studies (Slot & Winter, 2017a, 2017b), attributed to its direct influence on *A*_net_ by constraining CO_2_ diffusion into the leaf. Therefore, it is plausible that *g*_s_ and thermal time constant have opposing relationships with *T*_span_. The breadth of *T*_span_ is a direct consequence of the combined temperature sensitivity of stomatal and biochemical processes which limit photosynthesis above *T*_opt_ (Slot & Winter, 2017a). Whilst drought‐tolerant species had significantly lower *g*_sdiff_, *T*_optETR_ (indicative of the thermal optimum of biochemical processes) did not differ between drought‐tolerant and drought‐intolerant species. Therefore, the narrower *T*_span_ observed in drought‐intolerant species appears more likely a consequence of a more conservative stomatal strategy, as opposed to a greater thermal sensitivity of biochemical processes. Interestingly, species‐mean *T*_opt_ also did not differ between drought‐tolerant and drought‐intolerant species but coincided closely with site maximum annual air temperatures. These findings correspond to similar observations across other tropical forest sites and support the notion that photosynthetic performance is optimized according to growth temperatures, regardless of other plant functional traits, likely because of the advantages for maximizing carbon gain (Kumarathunge et al., 2019; Slot & Winter, 2017b; Tan et al., 2017). A lack of clear distinction between drought‐intolerant and drought‐tolerant species persisted across all other photosynthesis, respiration, and thermotolerance traits, suggesting that there is only limited coordination between drought and thermal sensitivity in adult tropical trees.

### 4.3 Differing TFE effects on drought‐tolerant and drought‐intolerant species thermal traits

After separating species by drought tolerance, there remained no TFE effect on any thermal traits in drought‐tolerant species, aside from *T*_50_ (Figure 4l). However, *R*_45_ and *Q*_10_ of drought‐intolerant species were lower in the TFE relative to the control by 31% and 29% respectively (Figure 4j,k). Whilst only marginally significant (*p* = 0.06; Figure 4e, Supporting Information: Figure S11, Supporting Information: Table S3) *g*_s*T*opt_ of drought‐intolerant species tended to downregulate in the TFE compared to the control, indicating that even at optimal temperatures for photosynthesis, drought‐intolerant species are tending towards a more water conservative stomatal strategy in the TFE. This will likely result in more pronounced increases in leaf temperatures (Fauset et al., 2018; Fauset et al., 2019) in the TFE for drought‐intolerant compared to drought‐tolerant species that showed no indication of downregulating *g*_s_ *_T_*_opt_. This potential exposure to higher leaf temperatures might explain why drought‐intolerant species exhibited some acclimation of physiological processes whilst drought‐tolerant species did not, for example, *R*_45_ and *Q*_10_ were reduced only in drought‐intolerant species. Alternatively, the fact that acclimation was only observed in drought‐intolerant species might suggest that they are generally more plastic in their response to stress compared to drought‐tolerant species.

In contrast to gas exchange traits that either did not change or indicated a slight thermal acclimation (in the case of *R*_45_ and *Q*_10_ in drought‐intolerant species), leaf thermotolerance was slightly weakened in both drought‐tolerant and intolerant species. Surprisingly, it was drought‐tolerant species that showed greater reductions in *T*_50_, both in terms of magnitude and the proportion of species with *T*_50_ reductions (Supporting Information: Figure S11). Leaf thermotolerances have been found to relate to maximum recorded leaf temperatures in tropical trees (Perez & Feeley, 2020). If drought‐intolerant species are more likely to experience critically high leaf temperatures due to a more conservative stomatal strategy, as our data suggest, then investment in maintenance of high thermotolerance thresholds is likely a higher priority for these species than for drought‐tolerant species. However, the underlying mechanism behind these differences remains unclear.

### 4.4 Wider context and conclusions

Our results suggest that, unlike short‐term drought that might pre‐condition plants for higher temperatures (Gauthier et al., 2014; Ghouil et al., 2003; Havaux et al., 1988; Ladjal et al., 2000; Sastry et al., 2018), sustained drought does not alter thermal sensitivity within moderate temperature ranges, but instead weakens trees’ ability to protect photosynthetic machinery under extreme temperatures. Whether or not this is a concern will depend on the frequency with which critical leaf temperatures are reached. Currently, maximum annual air temperature at this site is 33.8°C, so a reduction in *T*_50_ from 50 ± 0.3°C in the control to 48.5 ± 0.3°C under TFE conditions may seem irrelevant. However, leaf temperatures are known to exceed air temperatures by as much as 10°C–18°C (Doughty & Goulden, 2008; Fauset et al., 2018; Rey‐Sánchez et al., 2016). Accordingly, current maximum annual leaf temperatures may already approximate thermal thresholds during the hottest part of the year. Thus, even without any climate warming, the 1.5°C reduction in *T*_50_ of TFE trees could be sufficient to increase their risk of thermal damage. Whilst drought‐tolerant species appear to have a stronger weakening in thermotolerance compared to drought‐intolerant species in the TFE, it is important to contextualize this in terms of their thermal safety margins (the difference between *T*_50_ and maximum leaf temperatures), which may not be that different if drought‐tolerant species’ ability to maintain *g*_s_ rates at high temperatures translates to smaller leaf‐to‐air temperature differences. Indeed, it has been shown recently, on dryland plants, that high thermotolerance does not necessarily imply greater thermal safety but can signify greater hydraulic vulnerability and more acute exposure to heat stress (Cook et al., 2021). Whilst logistically challenging, continuous multi‐canopy measurements of in situ leaf temperatures would help resolve these complexities and elucidate the extent to which long‐term drought might increase the risk of leaf thermal damage in tropical forests.

## Supporting information

Supporting information.

Supporting information.

## Data Availability

The data that support the findings of this study are available in the Supporting Information: Dataset S1 of this manuscript.
