# Supplementary material for: Long‐term drought effects on the thermal sensitivity of Amazon forest trees
Source: Plant Cell Environ. 2022 Oct 20;46(1):185–98. doi: 10.1111/pce.14465 (PMC10092618; doi:10.1111/pce.14465)
Supplement: Supplementary file 2 — Supporting information. [file PCE-46-185-s002.pdf]

***New Phytologist* Supporting Information**

Article title: **Long-term drought effects on the thermal sensitivity of Amazon forest trees**

Authors: Emma M. Docherty, Emanuel Gloor, Daniela Sponchiado, Martin Gilpin, Carlos A. D. Pinto, Haroldo M. Junior, Ingrid Coughlin, Leandro Ferreira, João A. S. Junior, Antonio C. L. da Costa, Patrick Meir and David Galbraith

The following Supporting Information is available for this article:

Dataset S1 The data used for this manuscript

Fig. S1 Plots showing no effect of branch collection time on T_{opt} (a), A_{opt} (b), T_{span} (c), T_{max} (d) and R_{25} (e), and time delay between branch collection and initiation of respiration temperature response curves on R_{25} (f).

Fig. S2 Photosynthesis (A_{net}) temperature response curves for eight species in the control (blue) and TFE (red) plots at Caxiuanã.

Fig. S3 The relationship between leaf temperature and leaf-to-air vapour pressure deficit (VPD) (a), VPD and g_s (b), and g_s and A_{net} (c) during *A-T* response curve measurements in the control (blue dots) and the TFE (red dots) at Caxiuanã.

Fig. S4 Examples of *A-T* curves where differing equations provided the best fit and therefore the most realistic thermal trait extraction.

Fig. S5 Gardner-Altman plots comparing extracted parameters between equations used to fit *A-T* curves when AIC values were within 2 units of the most parsimonious equation.

Fig. S6 Stomatal conductance (g_s) temperature response curves for eight species in the control (blue) and TFE (red) plots at Caxiuanã.

Fig. S7 Electron transport rate (ETR) temperature response curves for eight species in the control (blue) and TFE (red) plots at Caxiuanã.

Fig. S8 Dark Respiration (R_{net}) temperature response curves for eight species in the control (blue) and TFE (red) plots at Caxiuanã.

Fig. S9 F_v/F_m temperature response curves for eight species in the control (blue) and TFE (red) plots at Caxiuanã.

Fig. S10 Boxplots showing the distributions of T_{opt} (a), A_{opt} (b), T_{span} (c), T_{max} (d), g_{sTopt} (e), g_{sTL46} (f), g_{sdiff} (g), T_{optETR} (h), R_{25} (i), R_{45} (j), Q_{10} (k), and T_{50} (l) in the control (blue) and the TFE (red) at Caxiuanã.

Fig. S11 Species average T_{opt} (a), A_{opt} (b), T_{span} (c), T_{max} (d), g_{sTopt} (e), g_{sTL46} (f), g_{sdiff} (g), T_{optETR} (h), R_{25} (i), R_{45} (j), Q_{10} (k), and T_{50} (l) in the control (blue) and the TFE (red) at Caxiuanã.

Table S1 Paired t-test results comparing extracted photosynthesis traits from the LI-6400 and LI-6800 LI-COR models.

Table S2 Results of mixed effect models of treatment (control vs TFE) and drought tolerance (drought-tolerant vs drought-intolerant) on thermal traits.

Table S3 Results of mixed effect models of treatment on thermal traits for species separated by drought tolerance.

Methods S1 Atypical A-T response curves

Methods S2 Atypical g_s-T response curves

Methods S3 Validity of leaf temperature measurements

Methods S4 Reasoning of measurement time period

Figure S1. Plots showing no effect of branch collection time on T_{opt} (a), A_{opt} (b), T_{span} (c), T_{max} (d) and R_{25} (e), and time delay between branch collection and initiation of respiration temperature response curves on R_{25} (f).

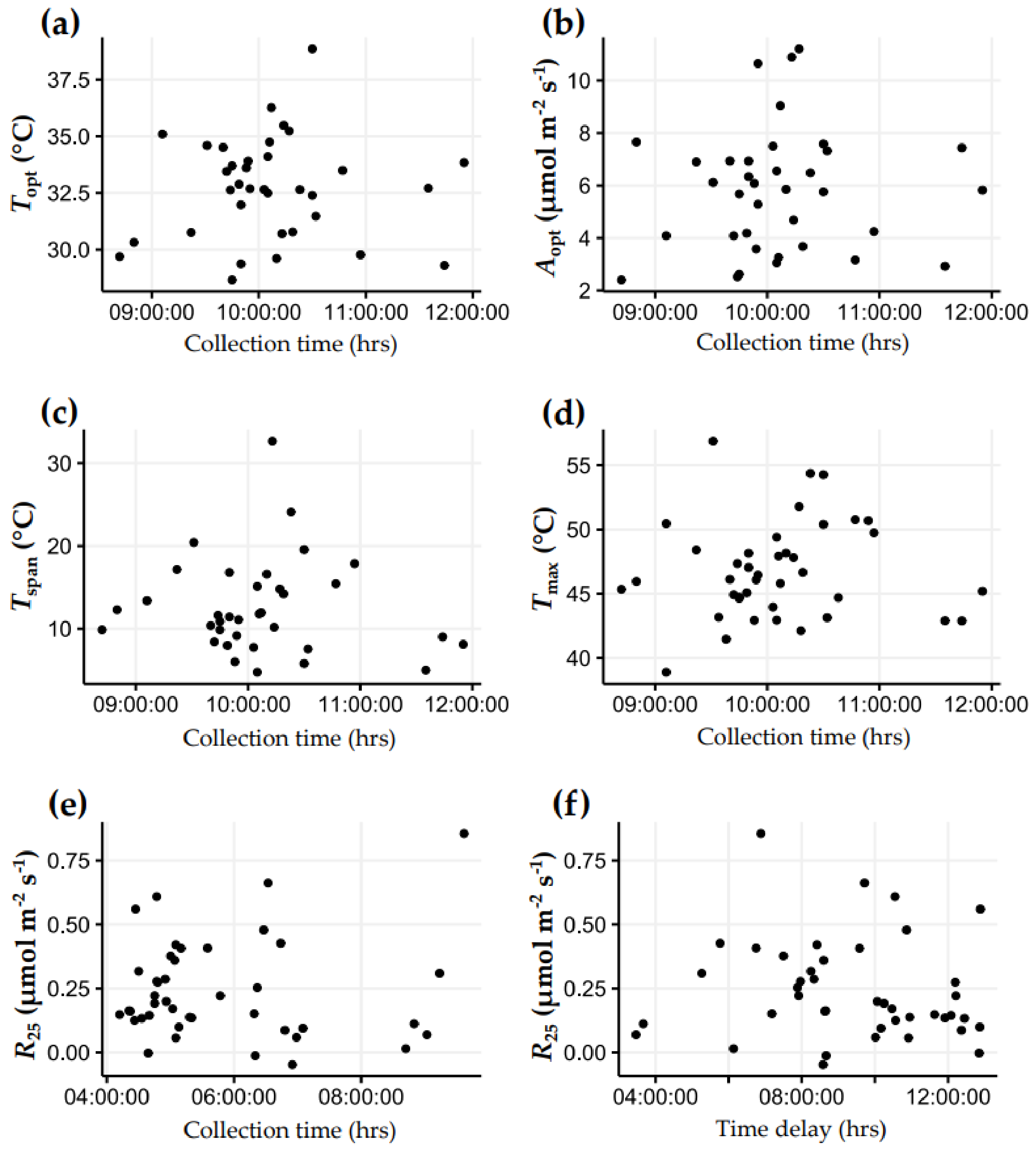

Figure S2. Photosynthesis (A_{net}) temperature response curves for eight species in the control (blue) and TFE (red) plots at Caxiuanã. Drought-intolerant species are on the left and drought-tolerant species on the right. Solid lines were fitted according to the equation (comparing equations 1-3) with the lowest AIC value for each individual *A*-*T* curve and used to extract T_{opt}, A_{opt} and T_{span}. Dashed lines were fitted according to the equation (comparing equations 1, 3 and 4) with the lowest AIC value for each individual *A*-*T* curve and used to extract T_{max}.

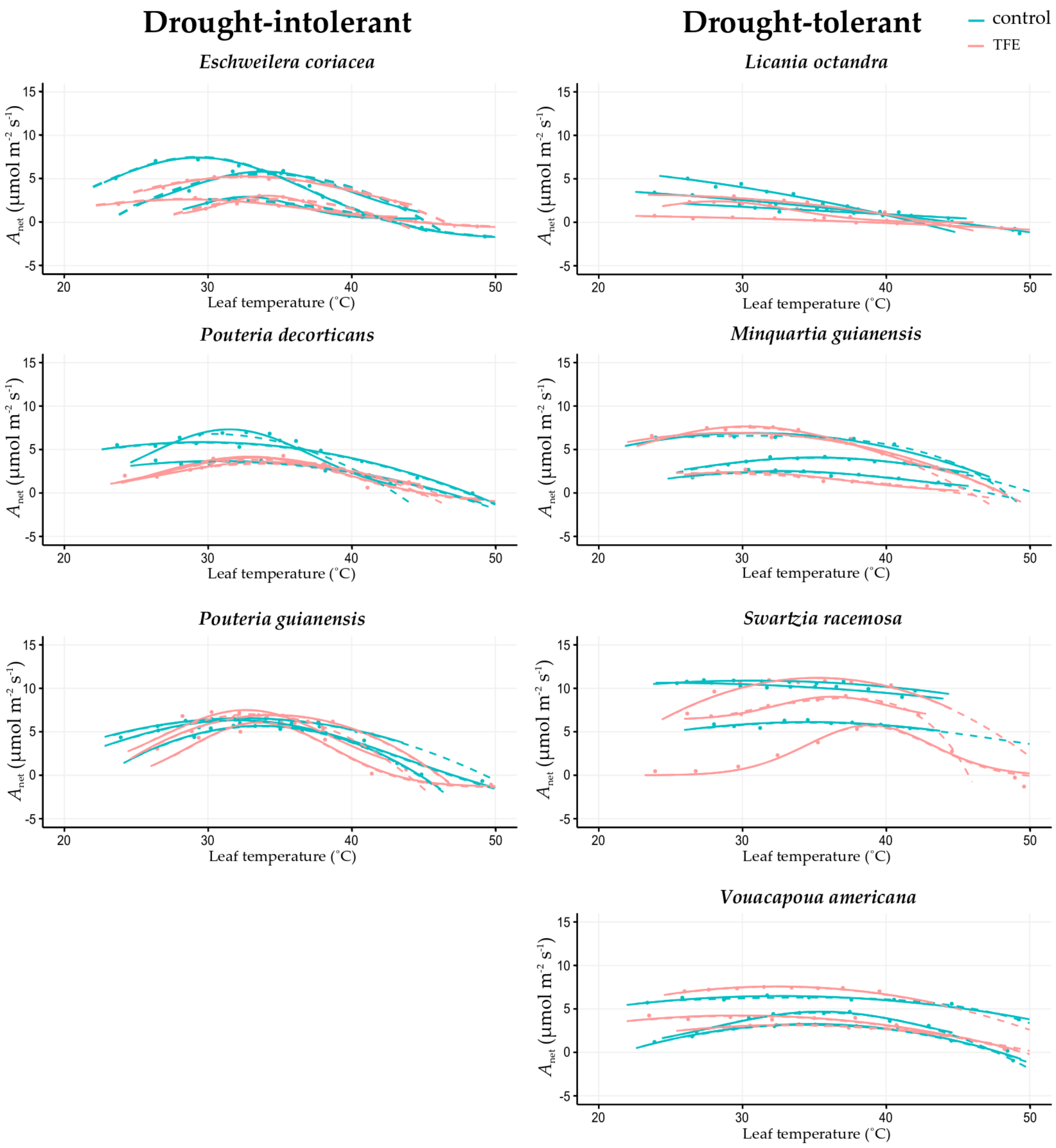

Figure S3. The relationship between leaf temperature and leaf-to-air vapour pressure deficit (VPD) (a), VPD and g_s (b), and g_s and A_{net} (c) during *A*-*T* response curve measurements in the control (blue dots) and the TFE (red dots) at Caxiuanã.

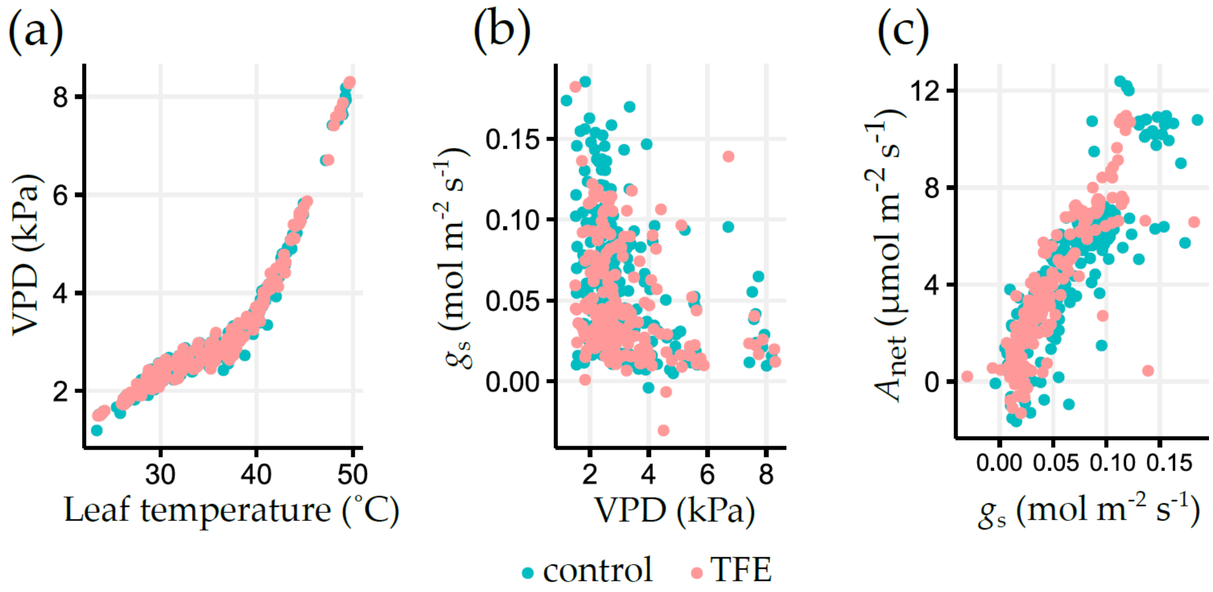

Figure S4. Examples of *A*-*T* curves where differing equations provided the best fit and therefore the most realistic thermal trait extraction. Showing *A*-*T* curves from Individual #1 (a-c), Individual #2 (d-f), Individual #3 (g-i), and Individual #4 (j-l), fit using Equation #1 (a, d, g, j), Equation #2 (b, e, h, k) and Equation #3 (c, f, i, l). The best fitting equations for individuals, based on lowest AIC value, are indicated by a red line.

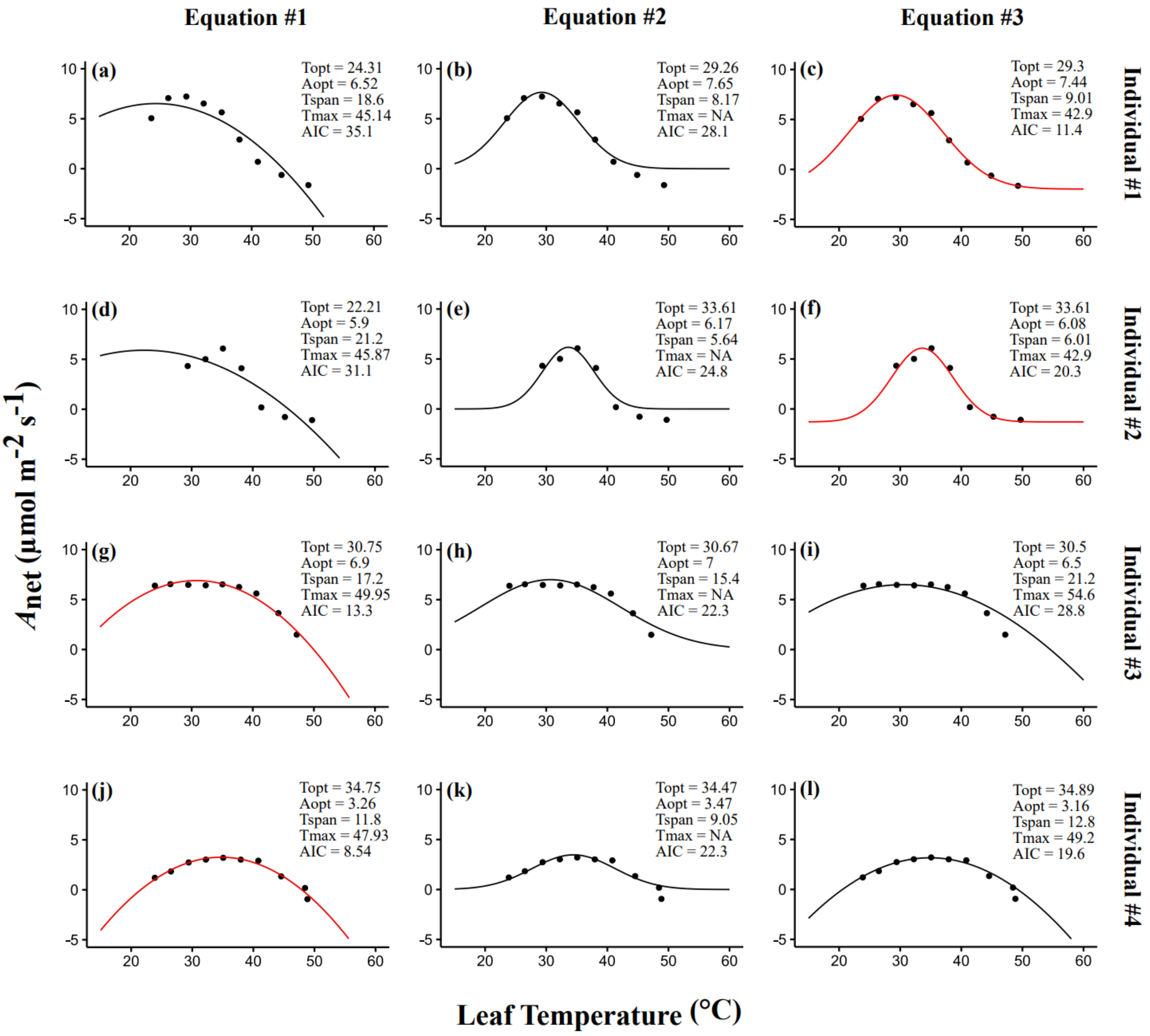

Figure S5. Gardner-Altman plots comparing extracted parameters between equations used to fit *A*-*T* curves when AIC values were within 2 units of the most parsimonious equation. The paired mean difference between models (black dot), and it's 95% confidence interval (thick black line), derived from 5000 nonparametric bootstrap resamples, is displayed on the right-hand aligned axis of each plot. Coloured lines represent individual *A*-*T* curves, showing the extracted T_{opt} values for equations 1-2 (a), 2-3 (b), and 1-3 (c), extracted A_{opt}, values for equations 1-2 (a), 2-3 (b), and 1-3 (c), extracted T_{span} values for equations 1-2 (d), 2-3 (e), and 1-3 (f), extracted T_{max} values for equations 1-4 (g), 1-3 (h). A comparison of extracted T_{max} values between equations 3-4 is not shown as these equations fit equally well for only one *A*-*T* curve and so it was not possible to plot, however, the extracted T_{max} values were still very similar at 48.2 °C and 48.1 °C respectively. Overall, equations that fit almost equally well produced very similar extracted traits with a maximum difference between equations of 0.5 °C, 0.4 μmol m^{-2} s^{-1}, 2.4 °C, and 0.3 °C for T_{opt}, A_{opt}, T_{span} and T_{max} respectively.

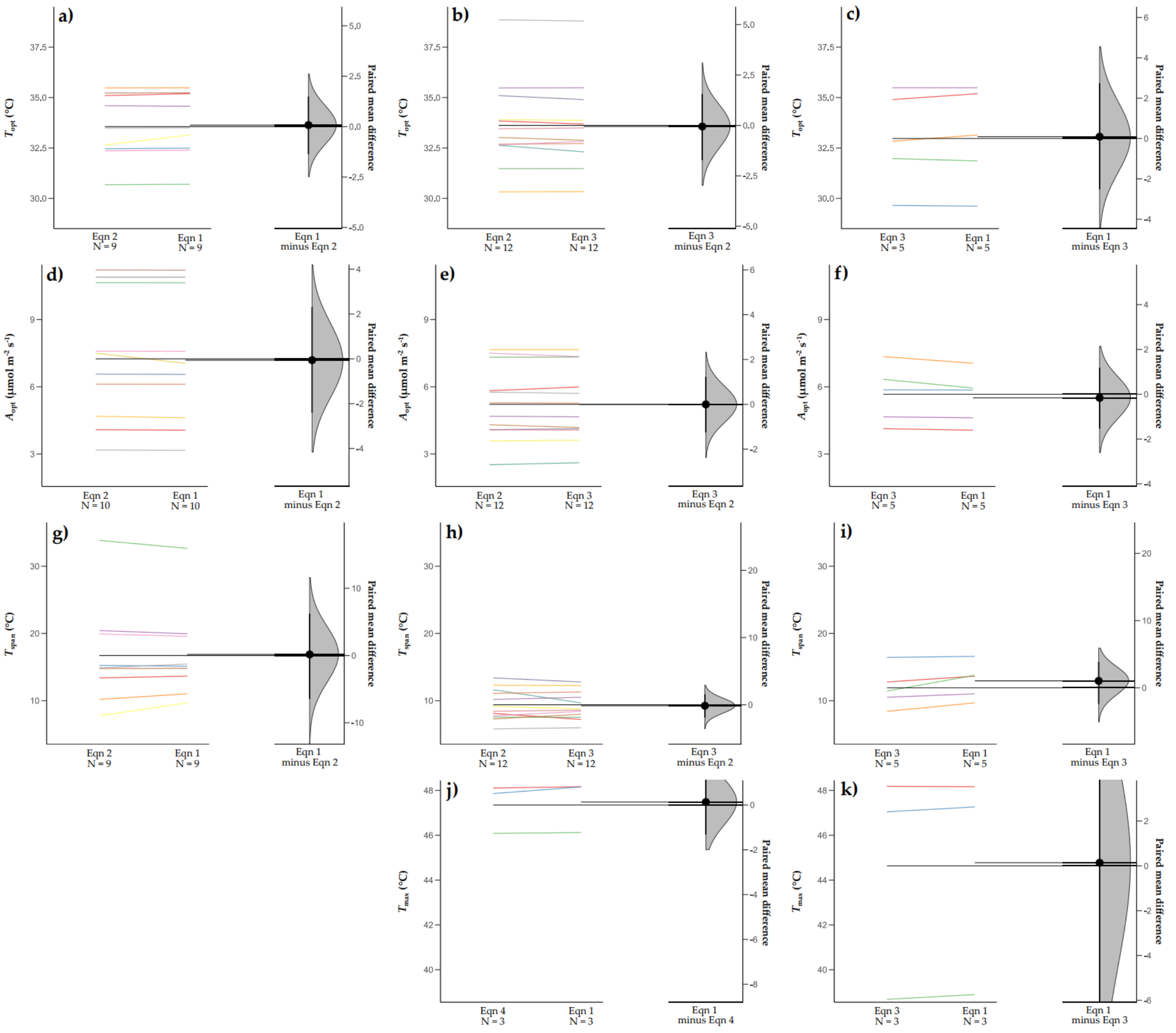

Figure S6. Stomatal conductance (g_s) temperature response curves for eight species in the control (blue) and TFE (red) plots at Caxiuanã. Drought-intolerant species are on the left and drought-tolerant species on the right. Solid lines were fitted for each individual curve according to the equation (comparing equations 1-4) with the lowest AIC value for each individual g_s-T curve and used to extract g_{sTopt} and g_{sTL46}.

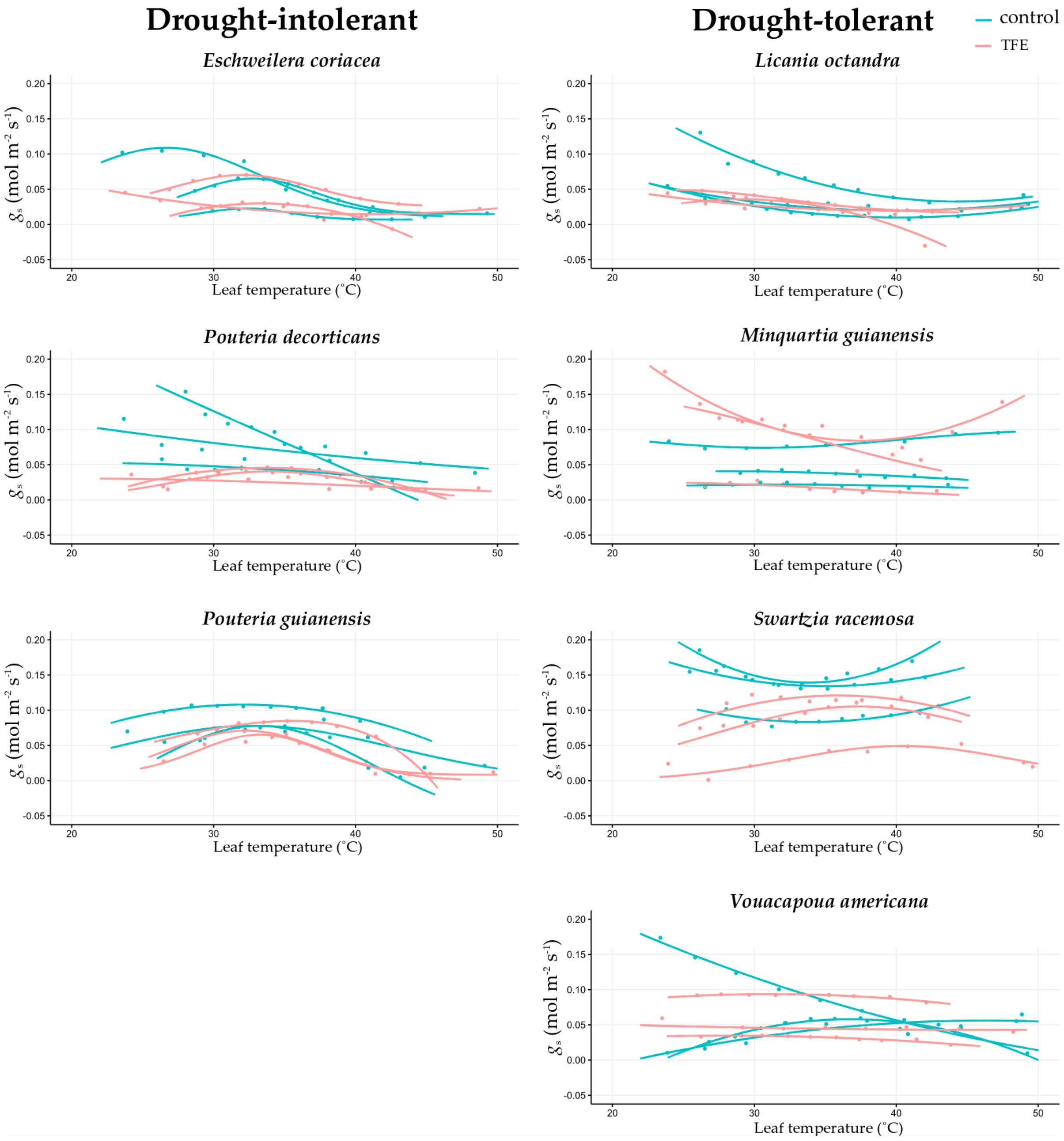

Figure S7. Electron transport rate (ETR) temperature response curves for eight species in the control (blue) and TFE (red) plots at Caxiuanã. Drought-intolerant species are on the left and drought-tolerant species on the right. Solid lines were fitted for each individual curve according to the equation (comparing equations 1-3) with the lowest AIC value for each individual *ETR-T* curve and used to extract T_{optETR}.

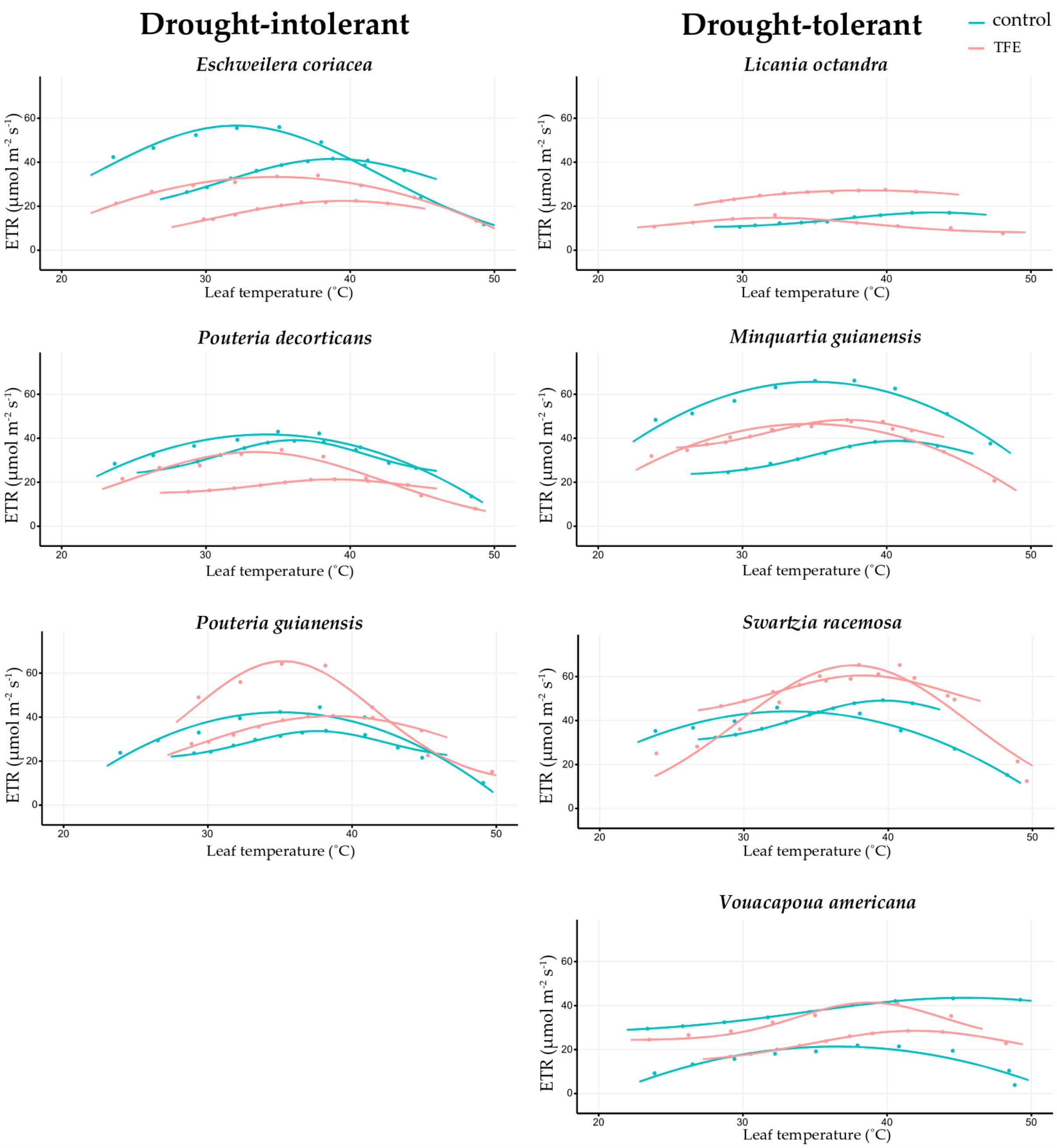

Figure S8. Dark Respiration (R_{net}) temperature response curves for eight species in the control (blue) and TFE (red) plots at Caxiuanã. Drought-intolerant species are on the left and drought-tolerant species on the right. Solid lines were fitted for each individual curve using a 3-point moving average polynomial regression and used to extract R_{25}, R_{45} and Q_{10}.

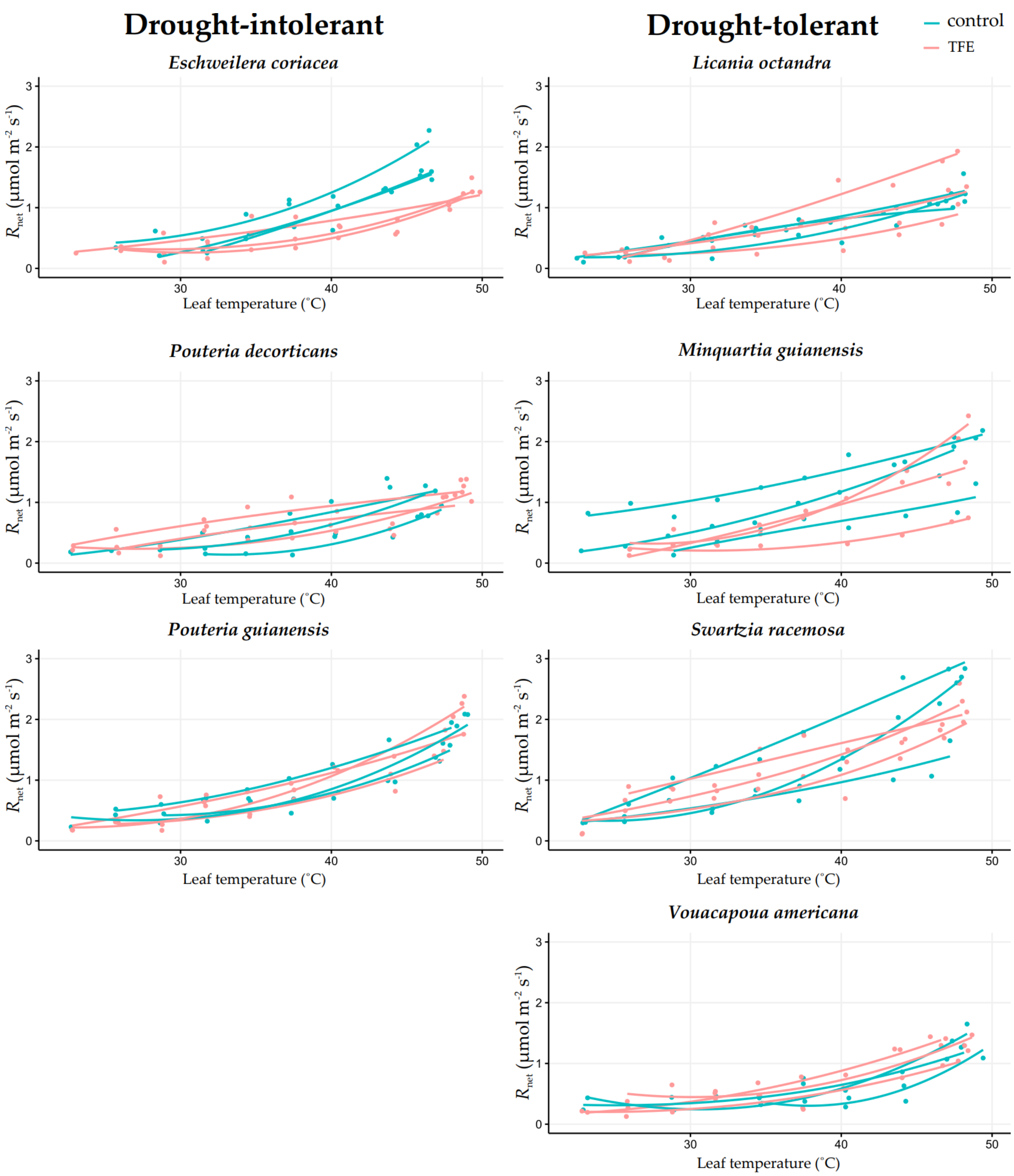

Figure S9. F_v/F_m temperature response curves for eight species in the control (blue) and TFE (red) plots at Caxiuanã. Drought-intolerant species are on the left and drought-tolerant species on the right. Solid lines were fitted for each individual curve according to equation 7 and used to extract T_{50}.

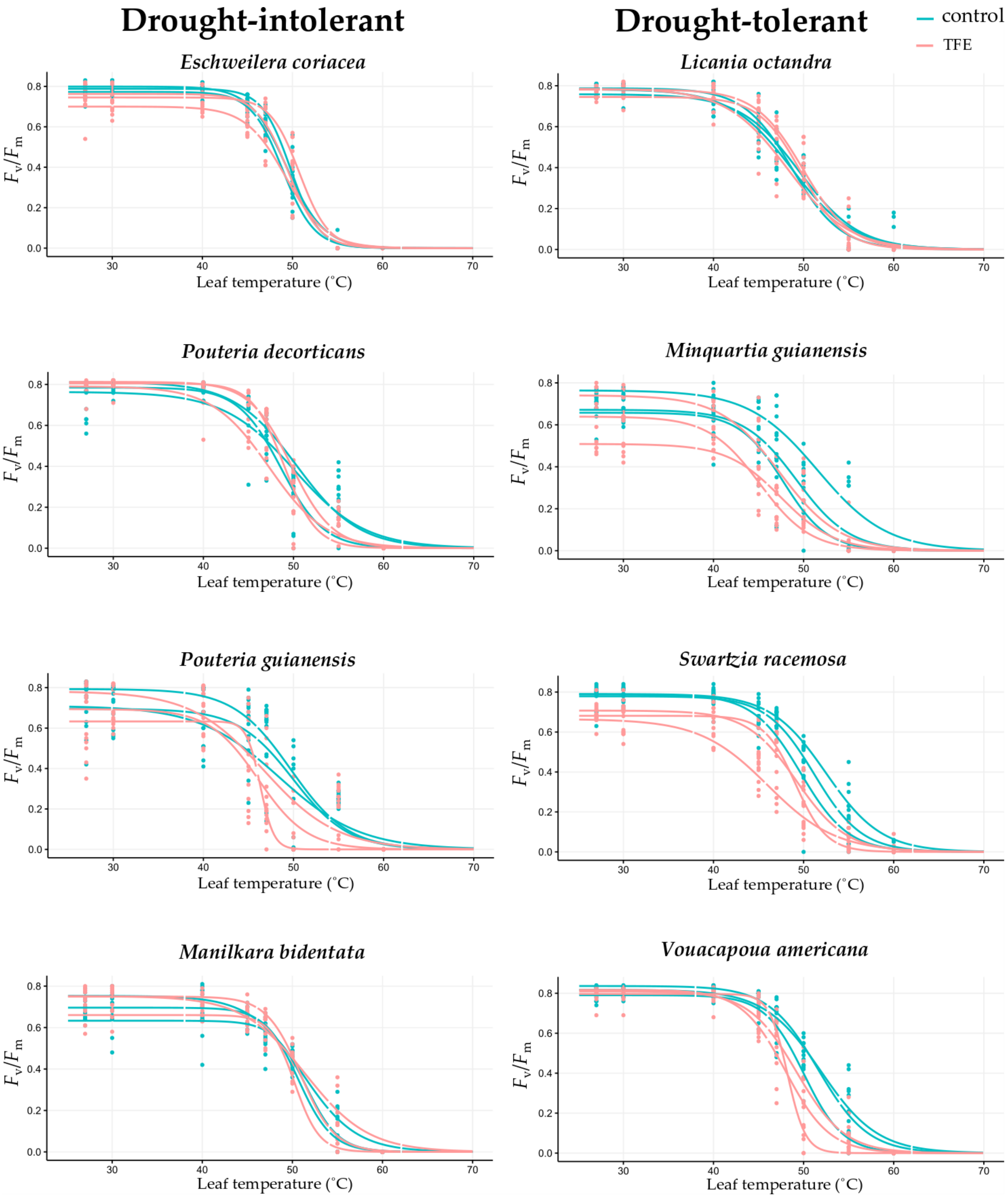

Figure S10. Boxplots showing the species mean distributions of T_{opt} (a), A_{opt} (b), T_{span} (c), T_{max} (d), g_{sTopt} (e), g_{sTL46} (f), g_{sdiff} (g), T_{optETR} (h), R_{25} (i), R_{45} (j), Q_{10} (k), and T_{50} (l) in the control (blue) and the TFE (red). Boxes show 25-75 percentiles, vertical lines show 10-90 percentiles, horizontal lines within boxes are medians, and points outside the boxes represent outliers. Blue stars represent significant differences between the control and TFE plots from mixed effects analysis (see Section 2.6).

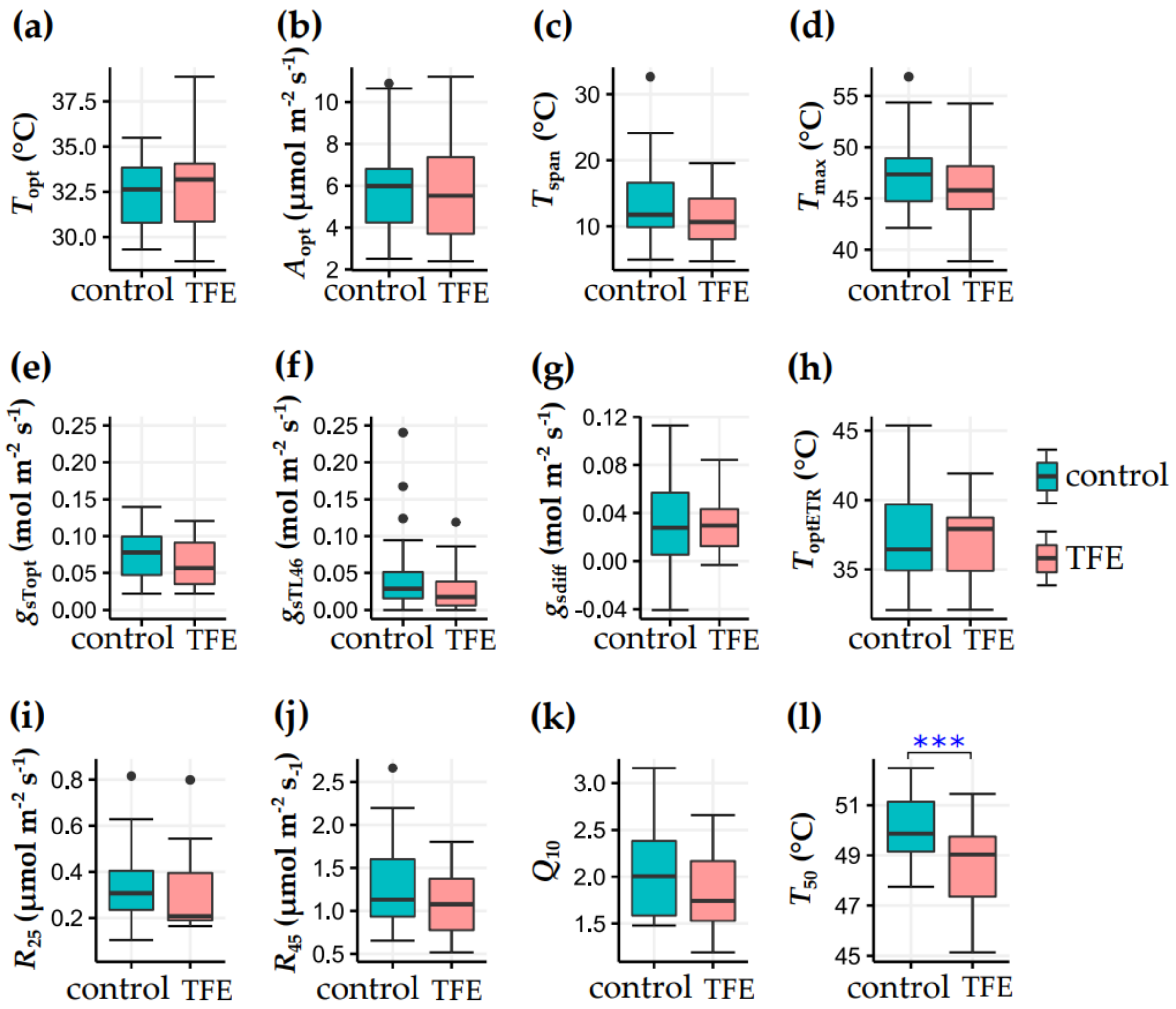

Figure S11. Species average T_{opt} (a), A_{opt} (b), T_{span} (c), T_{max} (d), g_{sTopt} (e), g_{sTL46} (f), g_{sdiff} (g), T_{optETR} (h), R_{25} (i), R_{45} (j), Q_{10} (k), and T_{50} (l) in the control (blue) and the TFE (red) at Caxiuanã. Error bars denote one standard error. Species codes: lioc = *Licania octandrai*, migu = *Minquartia guianensis*, swra = *Swartzia racemosa*, voam = *Vouacapoua americana*, esco = *Eschweilera coriacea*, pode = *Pouteria decorticans*, pogu = *Pouteria guianensis*, mabi = *Manilkara bidentata*.

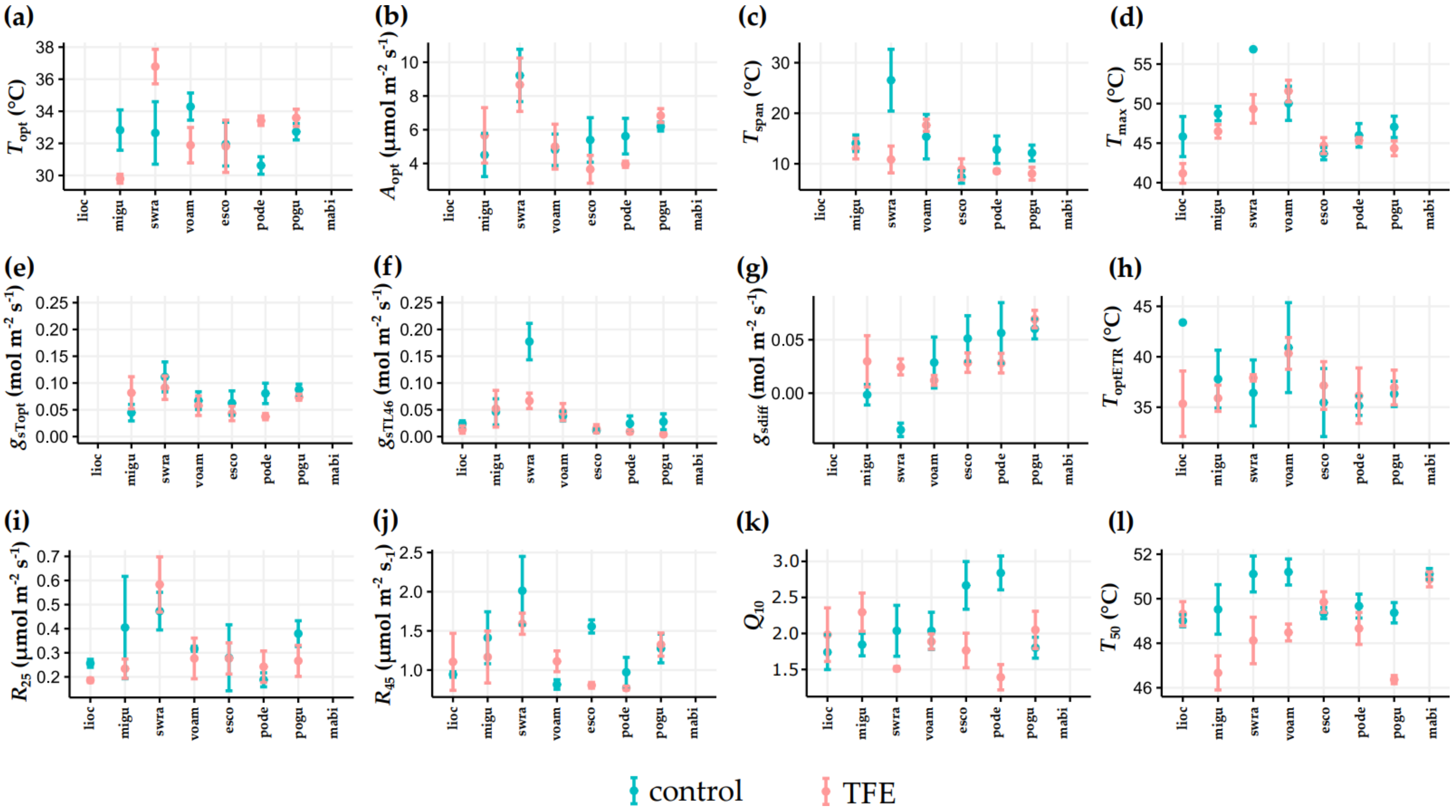

Table S1. Paired t-test results comparing extracted photosynthesis traits from the LI-6400 and LI-6800 LI-COR models, using the data from this study (Caxiuanã) and from a larger (unpublished) dataset of 87 tropical species.

**Trait**	**Dataset**	**Paired mean difference**	**95 % CI of the difference**		**t value**	**df**	***P*-value**
			**Lower**	**Upper**			
T_{opt}	Caxiuanã	1.152	-0.335	2.640	1.71	11	0.116
	Full	0.370	-0.232	0.972	1.23	57	0.223
A_{opt}	Caxiuanã	0.651	-0.886	2.187	0.92	13	0.377
	Full	0.354	-0.349	1.058	1.00	79	0.320
T_{span}	Caxiuanã	0.519	-2.642	3.680	0.36	11	0.725
	Full	0.292	-0.643	1.228	0.63	55	0.534
T_{max}	Caxiuanã	0.494	-1.104	2.091	0.66	16	0.522
	Full	0.063	-0.544	0.671	0.21	88	0.836

Table S2. Results of mixed effect models of treatment (control vs TFE) and drought tolerance (drought-tolerant vs drought-intolerant) on thermal traits (see Table 1 in main manuscript for descriptions and units). We show the coefficient values alongside the standard error (SE) for the intercept and fixed factors (treatment and drought tolerance) as well as the degrees of freedom (df), t value and the significance value (*P*) of the fixed factors. The marginal (including only fixed factors) and conditional r2 (including fixed and random (species) variables) are reported. n is the number of trait values included in the model and Sp is the number of species. The P-value of models with significant effects are shown in bold.

**Trait**	**Intercept**		**Fixed effect: Treatment**					**Fixed effect: Drought tolerance**					**Marginal r^2**	**Conditional r^2**	**n**	**Sp**
	**Coefficient**	**SE**	**Coefficient**	**SE**	**df**	**t value**	***P***	**Coefficient**	**SE**	**df**	**t value**	***P***				
T_{opt}	32.94	0.76	0.31	0.70	28.73	0.44	0.661	-0.75	0.92	5.60	-0.81	0.452	0.03	0.14	35	6
A_{opt}	6.47	0.90	-0.33	0.62	30.00	-0.52	0.605	-1.04	1.19	6.00	-0.87	0.418	0.06	0.34	36	6
$T_{span}{}^{i}$	2.79	0.10	-0.22	0.12	35.00	-1.86	0.071	-0.47	0.12	35.00	-4.08	**0.000**	0.37	0.37	35	6
T_{max}	49.15	1.31	-1.67	0.84	33.19	-2.00	0.054	-3.14	1.86	6.82	-1.69	0.137	0.21	0.53	40	7
$g_{sTopt}{}^{i}$	-2.66	0.17	-0.17	0.17	28.84	-0.98	0.337	-0.11	0.20	5.67	-0.53	0.614	0.04	0.09	35	6
$g_{sTL46}{}^{i}$	-3.01	0.33	-0.58	0.35	35.00	-1.66	0.105	-1.46	0.43	7.00	-3.37	**0.012**	0.31	0.36	42	7
$g_{sdiff}{}^{i}$	-3.02	0.14	0.19	0.16	35.00	1.23	0.226	0.58	0.16	35.00	3.69	**0.000**	0.30	0.30	35	6
$T_{optETR}{}^{ii}$	-	-	0.71	3.28	18.3	0.22	0.832	5.07	3.10	4.84	1.64	0.165	-	-	27	7
$R_{25}{}^{i}$	-1.15	0.15	-0.12	0.13	32.55	-0.89	0.378	-0.15	0.21	7.42	-0.75	0.478	0.04	0.23	39	7
R_{45}	1.35	0.15	-0.16	0.12	35.00	-1.33	0.193	-0.15	0.21	7.00	-0.72	0.495	0.06	0.30	42	7
Q_{10}	2.07	0.12	-0.30	0.15	42.00	-1.98	0.054	0.17	0.15	42.00	1.11	0.272	0.11	0.11	42	7
T_{50}	49.93	0.49	-1.50	0.36	40.00	-4.21	**0.000**	0.23	0.65	8.00	0.35	0.736	0.22	0.44	48	8

[i] Response variables that were log-transformed in order to deal with heteroscedasticity in error variance of the model.

[ii] Log transforming T_{optETR} did not fix heteroscedasticity and so an equivalent non-parametric Type III Walt F test with Kenward-Roger df was used to test the effect of treatment and drought tolerance on this parameter. Neither intercept coefficients and SE, nor r^2 values are produced for this test.

In order to log-transform g_{sTL46} and g_{sdiff} values were converted to positive values.

Table S3. Results of mixed effect models of treatment (control vs TFE) on thermal traits (see Table 1 in main manuscript for descriptions and units) for species separated by drought tolerance. We show the coefficient values alongside the standard error (SE) for the intercept and fixed factor (treatment) as well as the degrees of freedom (df), t value and the significance value (*P*). The marginal (including only fixed factor) and conditional r2 (including fixed and random (species) variables) are reported. n is the number of trait values included in the model and Sp is the number of species. The P-value of models with significant effects are shown in bold.

**Trait**	**Drought tolerance**	**Intercept**		**Fixed treatment effect**					**Marginal r^2**	**Conditional r^2**	**n**	**Sp**
		**Coefficient**	**SE**	**Coefficient**	**SE**	**df**	**t value**	***P***				
T_{opt}	Tolerant	33.48	1.09	-0.66	1.14	13.99	-0.57	0.580	0.02	0.22	17	3
	Intolerant	31.77	0.53	1.18	0.75	18.00	1.57	0.130	0.13	0.13	18	3
A_{opt}	Tolerant	6.18	1.20	0.27	1.04	15.00	0.26	0.800	0.00	0.35	18	3
	Intolerant	5.73	0.60	-0.92	0.65	15.00	-1.42	0.180	0.09	0.27	18	3
T_{span}[i]	Tolerant	2.80	0.13	-0.22	0.18	17.00	-1.26	0.230	0.09	0.09	17	3
	Intolerant	2.31	0.11	-0.21	0.15	18.00	-1.39	0.180	0.10	0.10	18	3
T_{max}	Tolerant	49.62	1.78	-2.47	1.33	18.23	-1.86	0.080	0.08	0.53	22	4
	Intolerant	45.57	0.61	-0.78	0.86	18.00	-0.91	0.380	0.05	0.05	18	3
g_{sTopt}[i]	Tolerant	-2.80	0.20	0.09	0.28	13.77	0.31	0.760	0.01	0.01	17	3
	Intolerant	-2.66	0.17	-0.40	0.20	15.00	-2.03	0.060	0.17	0.28	18	3
g_{sTL46}[i]	Tolerant	-3.02	0.41	-0.57	0.36	20.00	-1.58	0.129	0.07	0.39	24	4
	Intolerant	-4.46	0.43	-0.60	0.61	18.00	-0.98	0.340	0.05	0.05	18	3
g_{sdiff}[i]	Tolerant	-3.21	0.20	0.54	0.27	13.79	2.04	0.061	0.20	0.22	17	3
	Intolerant	-2.29	0.10	-0.13	0.12	15.00	-1.08	0.290	0.06	0.13	18	3
T_{optETR}	Tolerant[ii]	-	-	1.88	2.37	10.30	0.79	0.447	-	-	15	3
	Intolerant	35.64	0.93	1.11	1.32	12.00	0.84	0.420	0.06	0.06	12	3
R_{25}[i]	Tolerant	-1.13	0.19	-0.15	0.18	19.18	-0.83	0.418	0.02	0.31	23	4
	Intolerant	-1.31	0.15	-0.09	0.19	16.00	-0.47	0.646	0.01	0.01	16	3
R_{45}	Tolerant	1.30	0.19	-0.05	0.18	20.00	-0.29	0.777	0.00	0.28	24	4
	Intolerant	1.27	0.12	-0.30	0.13	15.00	-2.35	**0.033**	0.20	0.38	18	3
Q_{10}	Tolerant	1.91	0.12	0.00	0.17	24.00	0.03	0.977	0.00	0.00	24	4
	Intolerant	2.44	0.17	-0.70	0.24	18.00	-2.96	**0.008**	0.34	0.34	18	3
T_{50}	Tolerant	50.21	0.44	-2.06	0.56	20.00	-3.67	**0.002**	0.35	0.40	24	4
	Intolerant	49.88	0.59	-0.94	0.40	20.00	-2.35	**0.029**	0.10	0.58	24	4

[i] Response variables that were log-transformed in order to deal with heteroscedasticity in error variance of the model.

[ii] Log transforming T_{optETR} for drought-tolerant species did not fix heteroscedasticity and so an equivalent non-parametric Type III Walt F test with Kenward-Roger df was used to test for treatment differences for this parameter. Neither intercept coefficients and SE, nor r^2 values are produced for this test.

In order to log-transform g_{sTL46} and g_{sdiff} values were converted to positive values.

Methods S1. Atypical *A*-*T* response curves:

The natural shape of the *A*-*T* curves of *Licania octandra* (a negative slope with no obvious peak) and *Swartzia racemosa* (a wide curve with no obvious signs of decrease at high T_{leaf} in the control) were atypical (Fig. S2). Accordingly, it was not possible to extract T_{opt}, A_{opt}, T_{span}, g_{sTopt} and g_{sdiff} values for *L. octandra*. Likewise, it was not possible to extract T_{opt}, T_{span}, g_{sTopt} and g_{sdiff} for one *S. racemosa* individual and T_{max} for two *S. racemosa* individuals. The negative *A*-*T* response observed for *L. octandra* is uncommon, however, g_s rates were within the typical range of other species and were maintained by *L. octandra* individuals throughout the duration of temperature response curves (Fig. S6), providing no indication that the declining *A*-*T* response was due to branch excision artefacts.

Methods S2. Atypical g_s-*T* response curves:

Swartzia racemosa for example, adjusted its g_s-*T* response from a shallow U-shaped parabola in the control plot to a more typical bell-shaped parabola in the TFE (Fig. S6). The decrease in g_s as T_{leaf} increased towards T_{opt} (e.g., of *Swartzia racemosa* in the control) followed optimal stomatal behaviour, where g_s is proportional to A_{net}/(atmospheric CO_2/VPD) (Medlyn *et al.*, 2011), and was a result of unchanged A_{net} with increasing VPD (with T_{leaf} (Fig. S3)). Conversely, the rise in g_s above T_{opt} departed from optimal stomatal behaviour. This phenomenon has previously been observed in potted saplings (Ameye *et al.*, 2012; von Caemmerer & Evans, 2015; Slot *et al.*, 2016; Rogers *et al.*, 2017; Slot & Winter, 2017; Urban *et al.*, 2017), and 1 year-old (~6 metres tall) *Eucalyptus parramattensis* trees (Drake *et al.*, 2018), and is likely a component of some species response strategy to extreme temperatures, enabling leaf cooling to reduce/avoid thermal damage when water and hydraulic constraints are not limiting. Treatment-specific relationships between T_{leaf}, VPD, g_s and A_{net} did not differ (Fig. S3). Therefore, the adjustment from a shallow U-shaped parabola in the control plot to an bell-shaped parabola in the TFE, may then be linked to differences in the marginal water cost of plant carbon gain (Medlyn *et al.*, 2011), which is likely to be greater in the TFE where soil moisture is lower. Lower leaf water potentials (Ψ_{leaf}) in the TFE relative to the control (Bittencourt *et al.*, 2020; Rowland *et al.*, 2021) are also likely to be contributing to the g_s-*T* shift. Leaf water potential has been shown to influence g_s, such that for a given T_{leaf}, g_s decreases with Ψ_{leaf} (Li *et al.*, 2020). Therefore, when Ψ_{leaf} is more negative (i.e., under TFE conditions), both g_s and A_{net} are likely to be more sensitive to temperature.

Methods S3. Validity of leaf temperature measurements

We ensured LI-6400XT leaf temperature readings were as accurate as possible by zeroing the leaf temperature thermocouple (as described in the LI-6400XT manual) before starting each *A*-*T* curve, and ensuring leaf thermocouples were consistently in good contact with leaves, in both LI-COR models, by visually verifying that the thermocouple was accurately positioned just above the leaf gasket before each measurement and watching for a slight adjustment in measured leaf temperature upon clipping the leaf. In addition, each individual within a given species (per plot) were measured using a separate LI-COR. Since *A*-*T* curves were measured

on both LI-COR models for all species, we were able to compare all *A-T* traits extracted from the LI-6400XT and LI-6800, using paired t-tests (Table S1), confirming that LI-COR model caused no inherent bias in extracted *A-T* traits. This analysis was done for the data presented in this study as well as a larger dataset from 87 tropical broadleaf tropical tree species (unpublished), corroborating that both LI-COR models provide consistent thermal trait results.

Methods S4. Reasoning of measurement time period

It is possible that if measurements had been taken later in the dry season, potential thermal acclimation may have been more pronounced/observable due to the greater hydraulic and thermal stress that builds throughout the dry season in the TFE. However, the aim of our study was to determine if long-term reductions in soil moisture, as is the case in the TFE relative to the control throughout the whole year (Bittencourt *et al*., 2020), affects thermal sensitivity. Therefore, by conducting our measurements at the beginning of the dry season, succeeding the least stressful time of year, we could ensure that any differences observed were those resulting from the long-term soil water-stress as opposed to any short-term seasonal stress.

References

Ameye M, Wertin TM, Bauweraerts I, McGuire MA, Teskey RO & Steppe K 2012. The effect of induced heat waves on Pinus taeda and Quercus rubra seedlings in ambient and elevated CO 2 atmospheres. *New Phytologist* **196**: 448-461.

Bittencourt PR, Oliveira RS, da Costa AC, Giles AL, Coughlin I, Costa PB, Bartholomew DC, Ferreira LV, Vasconcelos SS & Barros FV 2020. Amazonia trees have limited capacity to acclimate plant hydraulic properties in response to long-term drought. *Global Change Biology* **26**: 3569-3584.

da Costa AC, Rowland L, Oliveira RS, Oliveira AA, Binks OJ, Salmon Y, Vasconcelos SS, Junior JA, Ferreira LV & Poyatos R 2018. Stand dynamics modulate water cycling and mortality risk in droughted tropical forest. *Global change biology* **24**: 249-258.

Drake JE, Tjoelker MG, Vårhammar A, Medlyn BE, Reich PB, Leigh A, Pfautsch S, Blackman CJ, López R & Aspinwall MJ 2018. Trees tolerate an extreme heatwave via sustained transpirational cooling and increased leaf thermal tolerance. *Global change biology* **24**: 2390-2402.

Li Y, Song X, Li S, Salter WT & Barbour MM 2020. The role of leaf water potential in the temperature response of mesophyll conductance. *New Phytologist* **225**: 1193-1205.

Medlyn BE, Duursma RA, Eamus D, Ellsworth DS, Prentice IC, Barton CV, Crous KY, De Angelis P, Freeman M & Wingate L 2011. Reconciling the optimal and empirical approaches to modelling stomatal conductance. *Global Change Biology* **17**: 2134-2144.

Rogers A, Medlyn BE, Dukes JS, Bonan G, Von Caemmerer S, Dietze MC, Kattge J, Leakey AD, Mercado LM & Niinemets Ü 2017. A roadmap for improving the representation of photosynthesis in Earth system models. *New Phytologist* **213**: 22-42.

Rowland L, Oliveira RS, Bittencourt PR, Giles AL, Coughlin I, Costa PdB, Domingues T, Ferreira LV, Vasconcelos SS & Junior JA 2021. Plant traits controlling growth change in response to a drier climate. *New Phytologist* **229**: 1363-1374.

Slot M, Garcia MN & Winter K 2016. Temperature response of CO2 exchange in three tropical tree species. *Functional Plant Biology* **43**: 468-478.

Slot M & Winter K 2017. Photosynthetic acclimation to warming in tropical forest tree seedlings. *Journal of Experimental Botany* **68**: 2275-2284.

Still CJ, Sibley A, Page G, Meinzer FC & Sevanto S, 2019. When a cuvette is not a canopy: A caution about measuring leaf temperature during gas exchange measurements. *Agricultural and Forest Meteorology* **279:** 107737.

Urban J, Ingwers M, McGuire MA & Teskey RO 2017. Stomatal conductance increases with rising temperature. *Plant signaling & behavior* **12**: e1356534.

von Caemmerer S & Evans JR 2015. Temperature responses of mesophyll conductance differ greatly between species. *Plant, Cell & Environment* **38**: 629-637.
